# Differential Habitat Use or Intraguild Interactions: What Structures a Carnivore Community?

**DOI:** 10.1371/journal.pone.0146055

**Published:** 2016-01-05

**Authors:** Matthew E. Gompper, Damon B. Lesmeister, Justina C. Ray, Jay R. Malcolm, Roland Kays

**Affiliations:** 1 Department of Fisheries & Wildlife Sciences, University of Missouri, Columbia, Missouri, United States of America; 2 USDA Forest Service, Pacific Northwest Research Station, Forestry Sciences Laboratory, Corvallis, Oregon, United States of America; 3 Wildlife Conservation Society Canada, Toronto, Ontario, Canada; 4 Faculty of Forestry, University of Toronto, Toronto, Ontario, Canada; 5 North Carolina Museum of Natural Sciences and North Carolina State University, Raleigh, North Carolina, United States of America; University of Missouri Kansas City, UNITED STATES

## Abstract

Differential habitat use and intraguild competition are both thought to be important drivers of animal population sizes and distributions. Habitat associations for individual species are well-established, and interactions between particular pairs of species have been highlighted in many focal studies. However, community-wide assessments of the relative strengths of these two factors have not been conducted. We built multi-scale habitat occupancy models for five carnivore taxa of New York’s Adirondack landscape and assessed the relative performance of these models against ones in which co-occurrences of potentially competing carnivore species were also incorporated. Distribution models based on habitat performed well for all species. Black bear (*Ursus americanus*) and fisher (*Martes pennanti*) distribution was similar in that occupancy of both species was negatively associated with paved roads. However, black bears were also associated with larger forest fragments and fishers with smaller forest fragments. No models with habitat features were more supported than the null habitat model for raccoons (*Procyon lotor*). Martens (*Martes americana*) were most associated with increased terrain ruggedness and elevation. Weasel (*Mustela* spp.) occupancy increased with the cover of deciduous forest. For most species dyads habitat-only models were more supported than those models with potential competitors incorporated. The exception to this finding was for the smallest carnivore taxa (marten and weasel) where habitat plus coyote abundance models typically performed better than habitat-only models. Assessing this carnivore community as whole, we conclude that differential habitat use is more important than species interactions in maintaining the distribution and structure of this carnivore guild.

## Introduction

There is an apparent contradiction between the strong interspecific interactions recorded in many detailed ecological studies and the assumption of spatial independence of taxa inherent in unified theories of biodiversity [1]. Indeed, the extent to which the spatial segregation of species represents ongoing interactions among species remains poorly understood. Co-occurring members of the mammalian order Carnivora provide a good opportunity to evaluate the degree to which species interact spatially. This is because many pairs of species demonstrate strong interference competition, which at its most extreme results in intraguild predation [2]. In a taxonomic order comprised of ca. 230 species, Palomares & Caro [3] documented 97 examples of interspecific killing, involving 54 victim species and 27 killer species; more recent research has increased these numbers (e.g., [4–6]).

While these data demonstrate that such interactions are both widespread in the order and can have severe consequences for interacting individuals, they do not reveal how commonly they occur in nature, nor if they result in broader effects on community structure. Nonetheless, it is increasingly assumed that the strength of intraguild interactions is sufficient to act as a driver of carnivore population distributions, and therefore carnivore community structure [2]. Given the potential for mammalian carnivores to directly and indirectly influence broader plant and animal communities, these interactions between carnivores could mediate trophic cascades [2,7–9].

Despite the potential importance of these interspecific interactions, they are typically not integrated with habitat associations when assessing the landscape ecology of species or communities [10]. Carnivores can have strong associations with particular habitat characteristics, and most efforts to understand the landscape ecology of these animals have been based on assessments of their distributions in relation to habitat features (e.g., [11–14]). Exceptions are those cases in which strong interactions are an *a priori* assumption; for example, where the carnivore taxa involved are closely related or interference competition is well documented [15–20]. These cases may not, however, represent the interactions typical of the broader carnivore community, for which the strength of competitive interactions among species pairs is often unclear. Simultaneous evaluations of species interactions and habitat preferences are needed to determine if responses to potential competitors reduces the use of putatively suitable habitat by a subordinate species due to the presence of a dominant species. Outcomes of predictive landscape models based solely on habitat may be biased if interspecific interactions are not considered.

We examined these issues for the carnivore community (7 focal species, ranging from *Mustela* spp. [weasels] to *Ursus americanus* [black bear]) occurring in and around the rural and forested landscapes of Adirondack State Park (ADK) in northern New York, USA. The ADK is the largest protected area in the contiguous United States (ca. 25,000 km^2^) and contains a broad range of habitat types, management units, human population densities, and resource extraction intensities. The carnivore community of this region has been subjected to significant anthropogenic perturbations typical of eastern North America: the larger native predators (*Puma concolor* [cougar], *Canis lupus* [gray wolf], and *Lynx canadensis* [lynx]) were driven to extinction in the late 1800’s and *C*. *latrans* (coyote) colonized the region in the 1950s and 1960s [21–22]. Our goal was to examine the extent to which competitive dynamics contributed to the local distributions of extant species in this community.

Our analysis is novel in that we evaluate the extent to which the presence of potentially competing taxa can serve as an additional source of information when creating predictive landscape models for target species based on habitat associations. Both theoretical and empirical studies of intraguild competition and predation suggest that this dynamic is often unidirectional, with interference occurring most intensely among similar-sized species and larger carnivores negatively influencing the smaller carnivores with which they directly interact [2,4]. Our approach was therefore to first construct multi-scale habitat models for each Adirondack carnivore species, and to then assess whether the inclusion of data on the occurrence of potentially competing carnivores improved the predictive strength of these habitat models. We were particularly interested in the role of *C*. *latrans*, as it is unclear to what extent this recent colonizer of the region influences the habitat use patterns of other forest carnivore species.

## Materials and Methods

### Study locale and survey techniques

Field work took place during 2000–2002 at 54 sites in and around Adirondack State Park (ADK), New York, USA. ADK is a mixture of state and private lands. Access for field work was granted by New York State Department of Environmental Conservation for state lands and individual landowners for private lands. We followed guidelines established by the American Society of Mammalogists Animal Care and Use Committee [23]. Our research protocol was approved by the Wildlife Conservation Society Animal Care and Use Committee and the New York State Department of Environmental Conservation permitting office (Permit No. LCP01-753). Survey methodologies and site details were described by Gompper et al. [24] and Kays et al. [12]. Sites were spread throughout the park and surrounding regions and were each separated by a minimum of 5 km. Sites had varying levels of anthropogenic modification, forest fragmentation, resource extraction (logging), and proximity to cities and agriculture. Collectively, these mid-elevation sites fell into three broad landscape categories: interior forest landscapes without active timber extraction (*n =* 31 sites), forest landscapes where active logging activities were occurring (*n =* 12 sites), and forest landscapes within or near suburban or agricultural landscapes (*n =* 11 sites).

At each site, we non-invasively surveyed the carnivore community at several sampling nodes situated along 5 km transects. Surveys were conducted using scat surveys (once per month for 3 months), camera traps (*n =* 3 per site left in place for 28–32 days [mean number of trap nights per transect = 88] and rebaited every 10 days) and track plates (*n =* 6 per site left in place for 11–15 days [mean number of trap nights per transect = 75] and checked and rebaited every 2–3 days) [24]. Scat surveys were effective for detecting *C*. *latrans*; DNA extracted at 31 of the study sites was used to determine the relationship between number of individuals per transect and scat abundance (*r*^*2*^ *=* 0.93), which was then used to estimate the number of individuals at the remaining sites [12]. Camera and track plate surveys yielded detection/non-detection information for other carnivores [24]. We combined data on the two regionally common weasel species (*Mustela erminea* and *M*. *frenata*), which were difficult to differentiate in our samples [24]. Several additional Carnivora (*Mephitis mephitis* [striped skunks], *Mustela vison* [minks], *Lontra canadensis* [river otter], *Urocyon cinereoargenteus* [grey foxes], *Vulpes vulpes* [red foxes], *Lynx rufus* [bobcats]) occur in the ADK, but are uncommon in the ADK forests where we worked, and were detected in our surveys too infrequently to include in our analyses. These species are generally easy to detect using our methods (e.g., [24–25]), underpinning their relative rarity in the region.

### Habitat data collection

To build habitat models for each species, we used a combination of local field-based habitat measurements and broad-scale metrics from remotely-sensed GIS layers (see S1 Table). Local measurements, taken at nine stations per transect and averaged for each transect, included: tree height, coarse woody debris volume, canopy openness, snag basal area, and proportion of coniferous trees (S1 Table; [12]). We quantified 17 remotely-sensed, landscape-scale habitat variables measuring forest types, open areas, edge habitats, snowfall, and anthropogenic disturbances using ArcGIS v.9 in buffers surrounding each transect at four scales: 0.5, 1, 5, and 10 km (S1 Table; [12]). We selected the larger scales as an approximate upper extreme for the radius of a *C*. *latrans* home range [26] and the two smaller scales to capture more fine–grained habitat relationships. GIS layers were from the NYS GIS Clearing House (http://www.nysgis.state.ny.us/), except for land use data that came from the New York State Gap Analysis Program (Cornell University, Ithaca, NY), and snowfall information, which was the average of snowfall within the buffers from the winters of 2002–3 and 2003–4 from the National Operational Hydrologic Remote Sensing Center. No remotely-sensed snow data were available from the exact years of our field surveys, so we consider these averaged data as an index of typical snowfall patterns for the region, and only used them for larger-scale analyses (5 km and 10 km buffers). We standardized all continuous covariates to z-scores prior to analysis, thereby interpreting model coefficients as the change in the log-odds ratio of occupancy relative to 1 standard deviation change in the covariate from its mean [27].

### Modeling techniques

**Fig 1:**
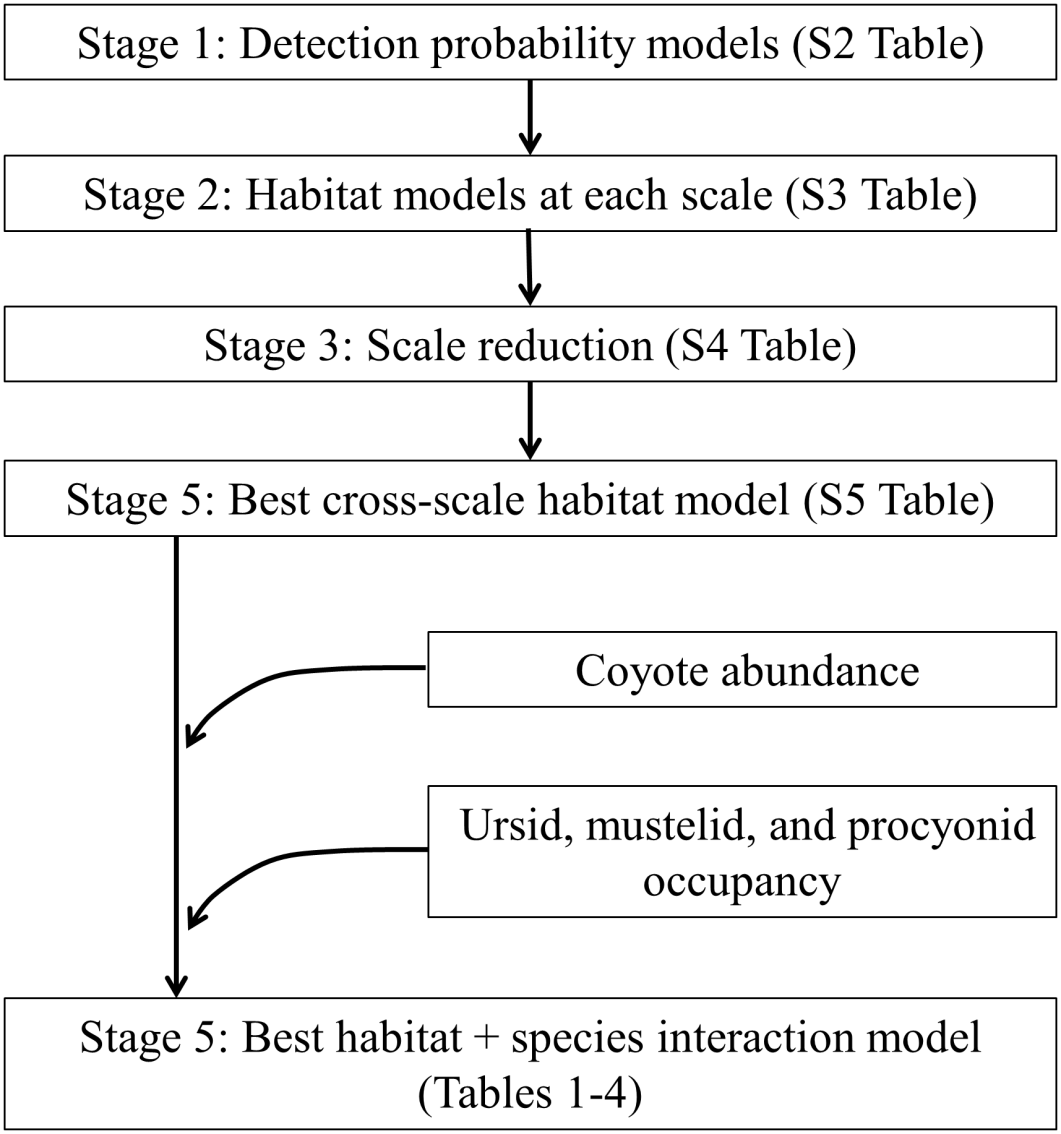
The modeling approach used to assess the manner in which the carnivore community in New York’s Adirondack landscape was predicted by combinations of habitat measures and species co-occurrence measures. For each species, detection probability models were generated (stage 1), with results of top models incorporated into models that contrasted how habitat features predicted species occurrences at multiple spatial scales (stage 2). We chose the most significant scale for each variable in most supported habitat models (stage 3) and then combined these into a single multi-scale habitat model (stage 4). Finally, to evaluate the importance of species interactions, we added species co-occurrence data to these multi-scale habitat models (stage 5).

Because detection of carnivores is imperfect, and likely varies among species and sites, we used the single-species, occupancy modeling technique described by MacKenzie et al. [28–29] and the program PRESENCE 3.0 [30] to determine the detectability and habitat factors that affected species distributions. Similarly to Lesmeister et al. [25], we used a multi-stage modeling approach that allowed us to first develop the best habitat model for each carnivore species, and then evaluated whether it was improved by adding interactions with other species. For example, if coyotes are significantly influencing the spatial distribution (and hence habitat use) of a species, then we would expect significant interaction in the model. That is, habitat use by the species would be expected to vary as a function of coyote abundance. We ranked models in each stage based on their Akaike’s Information Criterion values corrected for small sample sizes (AIC_c_) and model weights (*w*_*i*_) to select the most parsimonious model [29,31]. Because we had already created a habitat model for *C*. *latrans* abundance [12], we focused habitat modeling in this effort on *U*. *americanus*, *Martes (= Pekania) pennanti* [fisher], *M*. *americana* [marten], *Procyon lotor* [raccoon], and the two *Mustela* species. The first four modeling stages (Fig 1) involved building occupancy models for each species based on habitat variables collected at different scales, a robust approach that recognizes that species could respond to different variables at different scales [32], and an approach previously used for analyses of *C*. *latrans* [12]. In the first stage we modeled heterogeneity in detectability for each species to account for imperfect ability to detect a species during a survey. We did this by holding occupancy constant [ψ(.)] and used detection histories and survey-specific data to develop species-specific probabilistic models of survey detection probability (*p*). This allowed us to account for factors affecting our ability to detect species, including year and survey technique. We fit models of *p* with information on previous detections (if the species was detected at the site in ≥ 1 previous surveys), and method (track plate or camera trap). We used the most parsimonious species-specific *p* model (S2 Table) for all subsequent stages. We did not expect spatial autocorrelation to be a concern in our data; however, to address the possibility we fitted the Hines et al. [33] correlated detection model and compared it to the standard occupancy model. The AIC_c_ values of the correlated detection models were lower for only one of five species (fisher: ΔAICc = 2.59). For the remainder, correlation-detection models performed worse than the standard model. Therefore, we judged the standard occupancy model to be more appropriate for our dataset. In the second stage we created habitat models for each species at each scale, selecting habitat variables *a priori* based on the literature as important for these species (S3 Table). Stage 2 gave us 5 model sets for each species (i.e., one set for each of the four buffer sizes and one at the "local" scale, which included only the field-based measurements). In some cases the same habitat variable was important at different scales; therefore, our third modeling stage compared the most important habitat variables for a species across scales to determine the scale at which each habitat variable had the most support based on AIC_c_ scores (S4 Table). Specifically, for each variable in stage 3 that appeared in the 90% candidate set for a species at one or more of the landscape scales, we examined its model fit for the various landscape scales. The results of this cross-scale comparison were then used to select the most important habitat variables at the most appropriate scale to enter into stage 4, which developed the best cross-scale habitat models (S5 Table).

Finally, in a fifth stage we used occupancy and abundance estimates of other carnivores as potential explanatory variables in predicting occupancy for a focal species. There are known agonistic interactions for most of these dyads. We reasoned that the most intense shift in space use by the focal species would be detected by examining the influence of larger taxa on smaller taxa, and by examining influences among similar-sized species [4]. Thus, we hypothesized effects of *U*. *americanus* and *C*. *latrans* on smaller taxa and on one another, effects of *M*. *pennanti* and *P*. *lotor* on one another and on *M*. *americana* and *Mustela* spp., and effects of *M*. *americana* and *Mustela* spp. on one another.

We assessed the influence of the largest species in the study (*Ursus americanus*) on *C*. *latrans* by regressing estimated *U*. *americanus* occupancy on *C*. *latrans* site abundance. For all other interactions, we followed methods described by MacKenzie et al. [34] to estimate the magnitude of a species-presence effect on the probability of occurrence for each of the focal species. Because imperfect detection of target species could lead to misleading inferences about species co-occurrence patterns, we accounted for species-specific detection probabilities while modeling multispecies site occupancy [29, 34]. Thus, all models for a given taxa included top-ranked detection model parameters for both species in all ensuing co-occurrence models. Following MacKenzie et al. [29], we estimated the level of species co-occurrence as φ = (ψ^AB^)/ (ψ^A^ x ψ^B^), where ψ^A^ and ψ^B^ are probabilities of site occupancy by species A and B, respectively, and ψ^AB^ is the probability that a site is occupied by both species. If species co-occur randomly, φ = 1. Co-occurrence model sets included the most supported cross-scale habitat models of each potentially competing taxon. Using AIC_c_ values and model weights, we compared the most parsimonious co-occurrence models with the most parsimonious habitat-based models for each species to determine if the habitat model can be improved by considering species co-occurrences.

## Results

### Detection models

Overall occupancy and detection rates varied among taxa (*U americanus*: ψ = 0.640 ± 0.079, *p* = 0.390 ± 0.047; *M*. *pennanti*: ψ = 0.657 ± 0.073, *p* = 0.281 ± 0.030; *M*. *americana*: ψ = 0.229 ± 0.071, *p* = 0.183 ± 0.052; *P*. *lotor*: ψ = 0.556 ± 0.084, *p* = 0.223 ± 0.033; *Mustela* spp.: ψ = 0.404 ± 0.240, *p* = 0.059 ± 0.039). *Ursus americanus* was only detected using cameras and detectability was constant for the first three weeks (surveys 1–3; *p* = 0.411 ± 0.056), but differed for the fourth (*p* = 0.528 ± 0.092) and fifth week (*p* = 0.096 ± 0.065). Indeed, the model *p*(1–3, 4, 5) received 12 times more support than the next competing model (S2 Table). Detection of *M*. *pennanti* was positively influenced by previous detections at a site and there was a greater probability of detection by cameras (*p* = 0.350 ± 0.043) than track plates (*p* = 0.209 ± 0.036; S2 Table). *Martes pennanti* were more likely to be detected during a survey if ≥ 1 detection was recorded previously (*p* = 0.563 ± 0.056) compared to no previous detections (*p* = 0.101 ± 0.016). *Martes americana*, *P*. *lotor* and *Mustela* spp. all had greater probability of being detected with track plates (*p* = 0.309 ± 0.086, 0.271 ± 0.047, 0.105 ± 0.069, respectively) than cameras (*p* = 0.062 ± 0.037, 0.180 ± 0.037, 0.022 ± 0.020, respectively). Models accounting for differences in detectability by method received 1.6–6 times more support than the next competing model, with the weakest support occurring for *P*. *lotor* (S2 Table). With the exception of *M*. *pennanti*, we used the most parsimonious *p* model in subsequent stage models of occupancy. We were unable to model *M*. *pennanti* detection varying by capture history in subsequent stage models because of over-parameterization of models (K ≤ 9 for top detection probability models), so we used the null model *p*(.).

### *Ursus americanus* occupancy models

At each of the 5 spatial scales, several models for *U*. *americanus* had approximately equal support (S3 Table). At the local scale, increased basal area of snags had a positive influence on ψ and received the most support, whereas little improvement in model fit was observed with the addition of other field-based variables. The density of logging roads (an indicator of recent logging) and the average size of natural forest fragments had positive influence on *U*. *americanus* ψ at all larger scales. Conversely, the density of paved roads decreased ψ at all scales. The positive influence of forest cover decreased beyond the 0.5 km scale (S3 Table). The scale at which habitat features were most important to bears varied between the 5 parameters we evaluated (S4 Table). When combining the most important habitat variables across scales into one top model, we found that basal area of snags was in all models in the 90% confidence set and that forest cover and both types of roads were also in the most competitive models (S5 Table). The density of houses (-) and proportion of forest cover (+) were most influential at the smallest (0.5 km) scale, the proportion of deciduous forest (+) was most influential at the 5 km scale, and the density of paved (-) and logging roads (+), and the size of natural fragments (+) were most important at the largest (10 km) scale (S4 and S6 Tables). Adding data on local *C*. *latrans* abundance did not improve the fit of *U*. *americanus* habitat models (Table 1). The abundance of *C*. *latrans* also was not significantly influenced by the presence of *U*. *americanus* (F = 0.862, *p* = 0.357).

**Table 1:** *U*. *americanus* habitat models. Ranking of best *U*. *americanus* multi-scale habitat models in the 90% confidence set. We fit encounter history data from surveys at 54 sites in Adirondack Mountains, New York, USA to each candidate model set. See S1 Table for habitat variable descriptions.

Model	AIC_c_^a^	ΔAIC_c_	*w*^b^	K^c^	Deviance^d^
ψ (BASNAG + FORCOV0.5k + LOGRD10k)	250.36	0	0.219	7	233.93
ψ (BASNAG + PAVED10k + LOGRD10k)	251.04	0.68	0.156	7	234.61
ψ (FORCOV0.5k + BASNAG + PAVED10k + LOGRD10k)	251.05	0.69	0.155	8	231.85
ψ (BASNAG + FORCOV0.5k + DEC5k)	251.29	0.93	0.137	7	234.86
ψ (BASNAG + FORCOV0.5k + DEC5k + HEIGHT + CANOPEN)	251.63	1.27	0.116	9	229.54
ψ (BASNAG + FORCOV0.5k + LOGRD10k + COYOTE)	253.03	2.67	0.058	8	233.83
ψ (BASNAG + PAVED10k + LOGRD10k + COYOTE)	253.2	2.84	0.053	8	234
ψ (FORCOV0.5k + BASNAG + PAVED10k + LOGRD10k + COYOTE)	253.58	3.22	0.044	9	231.49

^a^ Akaike Information Criterion for small samples.

^b^ Model probability.

^c^ Number of model parameters.

^d^ Difference in -2Log(Likelihood) of the current model and -2log(Likelihood) of the saturated model as a measure of model fit.

### *Martes pennanti* occupancy models

**Fig 2:**
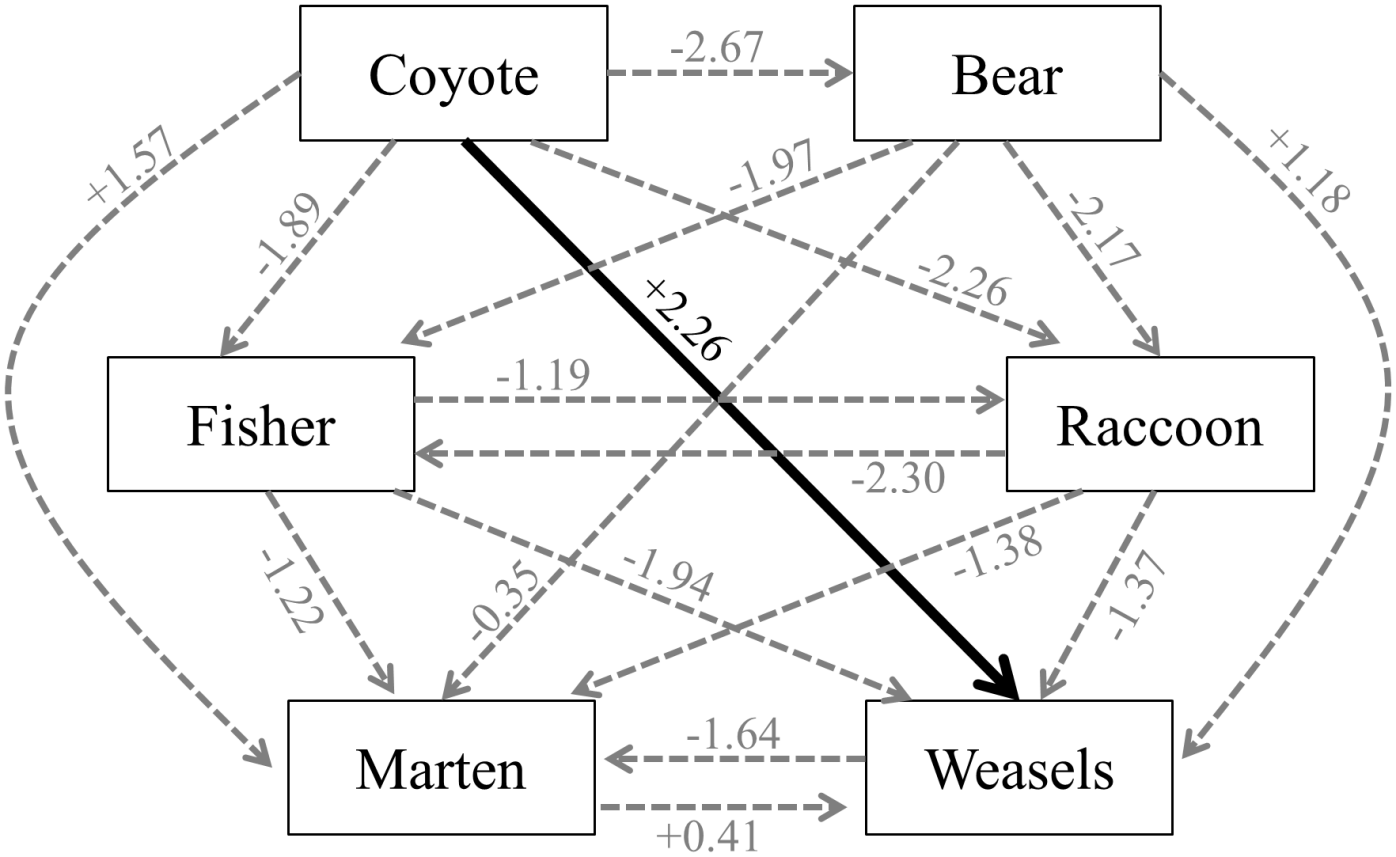
Extent to which adding interactions with another species improved (+) or worsened (-) the performance of the best habitat model (in terms of ΔAIC_c_ scores) for study sites in New York’s Adirondack landscape. In only one case (solid line) was a best model improved by > 2 AIC_c_ units through the inclusion of another carnivore species.

For *M*. *pennanti*, null occupancy [ψ(.)] was the top ranked local scale model and model ranking was strongly affected by the number of model parameters (S3 Table). At all larger scales, *M*. *pennanti* ψ was most influenced by average size of natural fragments (-) and density of houses (+) (S4 and S6 Tables). Forest cover had a positive influence on ψ at the 0.5 km scale only (S4 Table). Natural fragment size and paved road density most strongly affected ψ at the 10 km scale, whereas forest cover and house density were most important at the 0.5 km and 5 km scales, respectively (S4 Table). In the top cross-scale habitat model, density of houses (+) was the best predictor of *M*. *pennanti* ψ, with the addition of natural fragment size (-) and snag basal area (-) slightly improving model fit (S5 and S6 Tables). Co-occurrence model selection suggested that the addition of species interactions did not improve the fit of fisher habitat models (Table 2) as ΔAIC_c_ for the top models with *U*. *americanus*, *C*. *latrans*, and *P*. *lotor* were all ≥ 1.89 that of models solely derived from habitat metrics (Fig 2). Further, *M*. *pennanti* co-occurred randomly with *U*. *americanus* (φ = 0.950 ± 0.093) and *P*. *lotor* (φ = 1.012 ± 0.113).

**Table 2:** Fisher co-occurrence models. Co-occurrence model selection results in the 90% confidence set for *M*. *pennanti* with the inclusion of *U*. *americanus*, *C*. *latrans*, and *P*. *lotor*. We fit encounter history data from surveys at 54 sites in Adirondack Mountains, New York, USA to each candidate model set. Co-occurrence models were fit using important detection parameters and estimated occupancy, except *C*. *latrans* models which were fit using estimated abundance. See S1 Table for habitat variable descriptions.

Model	AIC_c_^a^	ΔAIC_c_	*w*^b^	K^c^	Deviance^d^
*Fisher models including black bear occupancy*					
ψ (NATFRAG10k + HOUSE5k)	530.42	0.00	0.319	8	511.22
ψ (HOUSE5k)	530.99	0.57	0.240	7	514.56
ψ (BASNAG + HOUSE5k)	531.94	1.52	0.149	8	512.74
ψ (HOUSE5k + BEAR)	532.39	1.97	0.119	8	513.19
ψ (NATFRAG10k + HOUSE5k + BEAR)	532.70	2.38	0.102	9	510.61
*Fisher models including coyote abundance*					
ψ (HEIGHT + HOUSE5k)	391.15	0.00	0.542	4	382.33
ψ (HEIGHT + HOUSE5k + COYOTE)	393.04	1.89	0.211	5	381.79
ψ (NATFRAG10k + HOUSE5k)	395.89	4.74	0.051	4	387.07
ψ (HOUSE5k)	395.90	4.75	0.050	3	389.42
ψ (HEIGHT + COYOTE)	396.97	5.82	0.030	4	388.15
ψ (BASNAG + HOUSE5k)	397.07	5.92	0.030	4	388.25
*Fisher models including raccoon occupancy*					
ψ (NATFRAG10k + HOUSE5k)	708.52	0.00	0.291	8	691.07
ψ (HOUSE5k)	708.54	0.02	0.288	7	693.42
ψ (HOUSE5k + BASNAG)	709.72	1.20	0.160	8	692.27
ψ (HOUSE5k + RACCOON)	710.82	2.30	0.092	8	693.37
ψ (NATFRAG10k + HOUSE5k + RACCOON)	710.82	2.30	0.092	9	690.98

^a^ Akaike Information Criterion for small samples.

^b^ Model probability.

^c^ Number of model parameters.

^d^ Difference in -2Log(Likelihood) of the current model and -2log(Likelihood) of the saturated model as a measure of model fit.

### *Procyon lotor* occupancy models

Although terrain ruggedness (+) and density of houses (-) were influential beyond the local scale (S6 Table), the null *P*. *lotor* occupancy model [ψ(.)] was the most supported model at all scales (S3 Table). In the cross-scale habitat model set, terrain ruggedness (+), house density (-), distance to nearest house (-), aspect (-), and snag basal area (-) were most influential variables of *P*. *lotor* ψ (S5 and S6 Tables). The addition of *C*. *latrans* abundance did not improve the fit of the *P*. *lotor* models over that of habitat-only models, and the influence of habitat was also more influential on *P*. *lotor* ψ than was the presence of *U*. *americanus* or *M*. *pennanti* (Table 3, Fig 2). *Procyon lotor* co-occurred randomly with *M*. *pennanti* (φ = 1.053 ± 0.115) and *U*. *americanus* (φ = 0.938 ± 0.112).

**Table 3:** Raccoon co-occurrence models. Co-occurrence model selection results in the 90% confidence set for *P*. *lotor* with the inclusion of *U*. *americanus*, *C*. *latrans*, and *M*. *pennanti*. We fit encounter history data from surveys at 54 sites in Adirondack Mountains, New York, USA to each candidate model set. Co-occurrence models were fit using important detection parameters and estimated occupancy, except *C*. *latrans* models which were fit using estimated abundance. See S1 Table for habitat variable descriptions.

Model	AIC_c_^a^	ΔAIC_c_	*w*^b^	K^c^	Deviance^d^
*Raccoon models including black bear occupancy*					
ψ (TRI0.5k + HOUSE5k + dtHOUSE)	479.79	0.00	0.379	10	457.52
ψ (TRI0.5k + BASNAG)	481.29	1.50	0.179	9	461.45
ψ (TRI0.5k + BASNAG + ASPECT0.5k)	481.50	1.71	0.161	10	459.23
ψ (TRI0.5k + HOUSE5k + dtHOUSE + BEAR)	481.96	2.17	0.128	11	457.21
ψ (TRI0.5k + BASNAG + BEAR)	483.68	3.89	0.054	10	461.41
*Raccoon models including coyote abundance*					
ψ (TRI0.5k + HOUSE5k + dtHOUSE)	312.90	0.00	0.315	6	299.11
ψ (TRI0.5k + BASNAG + ASPECT0.5k)	313.94	1.04	0.187	6	300.15
ψ (TRI0.5k + BASNAG)	314.04	1.14	0.178	5	302.79
ψ (TRI0.5k + BASNAG + COYOTE)	315.16	2.26	0.102	6	301.37
ψ (TRI0.5k + HOUSE5k + dtHOUSE + COYOTE)	315.53	2.63	0.085	7	299.1
ψ (TRI0.5k + BASNAG + ASPECT0.5k + COYOTE)	315.99	3.09	0.067	7	299.56
*Raccoon models including fisher occupancy*					
ψ (TRI0.5k + HOUSE5k + dtHOUSE)	698.98	0.00	0.340	9	679.14
ψ (TRI0.5k + HOUSE5k + dtHOUSE + FISHER)	700.17	1.19	0.188	10	677.90
ψ (TRI0.5k + BASNAG + ASPECT0.5k)	700.80	1.82	0.137	9	680.96
ψ (TRI0.5k + BASNAG)	700.94	1.96	0.128	8	683.49
ψ (TRI0.5k + BASNAG + FISHER)	701.56	2.58	0.094	9	681.72
ψ (TRI0.5k + BASNAG + ASPECT0.5k + FISHER)	701.94	2.96	0.077	10	679.67

^a^ Akaike Information Criterion for small samples.

^b^ Model probability.

^c^ Number of model parameters.

^d^ Difference in -2Log(Likelihood) of the current model and -2log(Likelihood) of the saturated model as a measure of model fit.

### *Martes americana* occupancy models

At the local scale, *M*. *americana* ψ increased with higher basal area of snags, but decreased with increases in canopy openness and tree height (S3 Table). Beyond the local scale, terrain ruggedness (+) and elevation (+) had the greatest influence on *M*. *americana* ψ (S4 and S6 Tables). Forest cover (+), elevation, terrain ruggedness, and proportion of conifer forest (+) were most influential at the 10 km scale, while snowfall (+) and size of natural fragment (+) were most influential at the 5 km scale (S4 and S6 Tables). In the cross-scale habitat model set for *M*. *americana*, terrain ruggedness was the most important variable (S5 Table). However, to different degrees, the volume of coarse woody debris (+), snag basal area, natural fragment size, and canopy openness improved model fit (S5 and S6 Tables).

*Martes americana* ψ was marginally and negatively influenced by *C*. *latrans*, as evidenced by the improved fit of habitat models with the addition of estimated *C*. *latrans* abundance (Table 4; ΔAIC_c_ = 1.57; β ± SE = -0.581 ± 0.255). The influence of habitat was more influential on *M*. *americana* ψ than was the presence of any of the other four co-occurring carnivores (Table 4) as ΔAIC_c_ for the top habit-only models was 0.35–1.64 improved over models that included both the habitat metrics and other members of the carnivore community (Fig 2). *Martes americana* ψ was higher at sites occupied by *U*. *americanus* than at sites not occupied by the species (0.305 ± 0.089 vs. 0.030 ± 0.069), and *M*. *americana* co-occurred randomly with *M*. *pennanti* (φ = 0.976 ± 0.219), *P*. *lotor* (φ = 1.189 ± 0.521) and with *Mustela* spp. (φ = 1.357 ± 0.757).

**Table 4:** Marten co-occurrence models. Co-occurrence model selection results in the 90% confidence set for *M*. *americana* with the inclusion of *U*. *americanus*, *C*. *latrans*, *M*. *pennanti*, *P*. *lotor*, and *Mustela* spp. We fit encounter history data from surveys at 54 sites in Adirondack Mountains, New York, USA to each candidate model set. Co-occurrence models were fit using important detection parameters and estimated occupancy, except *C*. *latrans* models which were fit using estimated abundance. See S1 Table for habitat variable descriptions.

Model	AIC_c_^a^	ΔAIC_c_	*w*^b^	K^c^	Deviance^d^
*Marten models including black bear occupancy*					
ψ (VOLCWD + TRI10k)	350.88	0.00	0.237	9	328.79
ψ (TRI10k + BEAR)	351.23	0.35	0.199	9	329.14
ψ (TRI10k)	351.60	0.72	0.166	8	332.40
ψ (TRI10k + BASNAG)	351.72	0.84	0.160	9	329.63
ψ (VOLCWD + TRI10k + BEAR)	351.95	1.07	0.139	10	326.83
ψ (TRI10k + BASNAG + BEAR)	352.55	1.67	0.103	10	327.43
*Marten models including coyote abundance*					
ψ (TRI10k + NATFRAG5k + COYOTE)	112.13	0.00	0.246	6	98.34
ψ (TRI10k + COYOTE)	113.18	1.05	0.146	5	101.93
ψ (VOLCWD + TRI10k)	113.70	1.57	0.112	5	102.45
ψ (VOLCWD + TRI10k + COYOTE)	113.87	1.74	0.103	6	100.08
ψ (TRI10k + BASNAG)	114.45	2.32	0.077	5	103.20
ψ (TRI10k + BASNAG + COYOTE)	114.58	2.45	0.072	6	100.79
ψ (TRI10k)	114.68	2.55	0.069	4	105.86
ψ (TRI10k + NATFRAG5k)	114.70	2.57	0.068	5	103.45
ψ (TRI10k + CANOPEN)	115.15	3.02	0.054	5	103.90
*Marten models including fisher occupancy*					
ψ (VOLCWD + TRI10k)	514.06	0.00	0.294	8	496.61
ψ (BASNAG + TRI10k)	514.84	0.78	0.199	8	497.39
ψ (TRI10k)	515.08	1.02	0.177	7	499.96
ψ (VOLCWD + TRI10k + FISHER)	515.28	1.22	0.160	9	495.44
ψ (BASNAG + TRI10k + FISHER)	516.27	2.21	0.097	9	496.43
*Marten models including raccoon occupancy*					
ψ (VOLCWD + TRI10k)	432.59	0.00	0.299	8	415.14
ψ (BASNAG + TRI10k)	433.24	0.65	0.216	8	415.79
ψ (TRI10k)	433.67	1.08	0.174	7	418.55
ψ (BASNAG + TRI10k + RACCOON)	433.97	1.38	0.15	9	414.13
ψ (VOLCWD + TRI10k + RACCOON)	434.75	2.16	0.102	9	414.91
*Marten models including weasel occupancy*					
ψ (BASNAG + TRI10k)	204.50	0.00	0.351	7	189.38
ψ (TRI10k)	205.52	1.02	0.211	6	192.69
ψ (BASNAG + TRI10k + WEASEL)	206.14	1.64	0.154	8	188.69
ψ (VOLCWD + TRI10k)	206.41	1.91	0.135	7	191.29
ψ (TRI10k + WEASEL)	207.08	2.58	0.097	7	191.96

^a^ Akaike Information Criterion for small samples.

^b^ Model probability.

^c^ Number of model parameters.

^d^ Difference in -2Log(Likelihood) of the current model and -2log(Likelihood) of the saturated model as a measure of model fit.

### *Mustela* occupancy models

The ψ of *Mustela* spp.was higher at sites with greater proportions of coniferous forest at the local scale and with higher levels of forest cover and deciduous forest at broader scales (S3 and S6 Tables). Multiple scales were represented in the most influential variables of ψ (S4 Table). Overall, the proportion of deciduous cover (+) was identified as the most important predictor of *Mustela* ψ (S5 and S6 Tables).

In three of six scenarios, the addition of data on co-occurring carnivores improved the fit of the *Mustela* spp. ψ models (Fig 2). The addition of *C*. *latrans* abundance improved the fit of *Mustela* ψ models over that of habitat-only models (ΔAIC_c_ = 2.26; Fig 2). The top model included both the proportion of deciduous cover within 1 km and the abundance of coyotes (Table 5), and all top models included coyote abundance. *Mustela* ψ was negatively influenced by higher levels of *C*. *latrans* abundance (β ± SE = -0.080 ± 0.124). The addition of *U*. *americanus* and *M*. *americana* presence also marginally improved the fit of *Mustela* spp. ψ models over top habitat-only models (Table 5). However, *U*. *americanus* and *Mustela* spp. co-occurred greater than expected by chance (φ = 1.405 ± 0.247), and *M*. *americana* and *Mustela* spp. co-occurred randomly with respect to one another (φ = 1.184 ± 0.210). Although *Mustela* spp. co-occurred more than expected with *M*. *pennanti* (φ = 1.400 ± 0.208), the latter did not improve the fit of *Mustela* spp. habitat ψ models (Table 5). Incorporation of *P*. *lotor* into *Mustela* spp. ψ models did not improve the fit of the models over that observed for habitat-only models, and the two taxa co-occurred randomly with respect to one another (φ = 1.152 ± 0.314).

**Table 5:** Weasel co-occurrence models. Co-occurrence model selection results in the 90% confidence set for *Mustela* spp with the inclusion of *U*. *americanus*, *C*. *latrans*, *M*. *pennanti*, *P*. *lotor*, and *M*. *americana*. We fit encounter history data from surveys at 54 sites in Adirondack Mountains, New York, USA to each candidate model set. Co-occurrence models were fit using important detection parameters and estimated occupancy, except *C*. *latrans* models which were fit using estimated abundance. See S1 Table for habitat variable descriptions.

Model	AIC_c_^a^	ΔAIC_c_	*w*^b^	K^c^	Deviance^d^
*Weasel models including bear occupancy*					
ψ (DEC1k + PROPSW + BEAR)	337.72	0.00	0.503	10	315.45
ψ (DEC1k + PROPSW)	338.90	1.18	0.279	9	319.06
ψ (DEC1k + BEAR)	340.70	2.98	0.113	9	320.86
ψ (DEC1k)	342.25	4.53	0.052	8	324.80
*Weasel models including coyote abundance*					
ψ (DEC1k + COYOTE)	89.66	0.00	0.359	5	79.07
ψ (DEC1k + PROPSW + COYOTE)	90.36	0.70	0.253	6	77.53
ψ (DEC1k + SNOW10k + COYOTE)	91.52	1.86	0.142	6	78.69
ψ (DEC1k)	91.92	2.26	0.116	4	83.53
ψ (DEC1k + PROPSW)	92.73	3.07	0.077	5	82.14
*Weasel models including fisher occupancy*					
ψ (DEC1k + PROPSW)	494.52	0.00	0.332	7	480.52
ψ (DEC1k)	495.13	0.61	0.245	6	483.13
ψ (DEC1k + PROPSW + FISHER)	496.46	1.94	0.126	8	480.46
ψ (DEC1k + SNOW10k)	496.72	2.20	0.110	7	482.72
ψ (DEC1k + FISHER)	496.96	2.44	0.098	7	482.96
*Weasel models including raccoon occupancy*					
ψ (DEC1k + PROPSW)	411.42	0.00	0.562	9	391.58
ψ (DEC1k + PROPSW + RACCOON)	412.79	1.37	0.284	10	390.52
ψ (DEC1k)	415.49	4.07	0.074	8	398.04
*Weasel models including marten occupancy*					
ψ (DEC1k + PROPSW + MARTEN)	203.44	0.00	0.270	8	185.99
ψ (DEC1k + PROPSW)	203.85	0.41	0.220	7	188.73
ψ (DEC1k + MARTEN)	204.36	0.92	0.170	7	189.24
ψ (DEC1k)	204.57	1.13	0.153	6	191.74
ψ (DEC1k + SNOW10k)	205.44	2.00	0.099	7	190.32

^a^ Akaike Information Criterion for small samples.

^b^ Model probability.

^c^ Number of model parameters.

^d^ Difference in -2Log(Likelihood) of the current model and -2log(Likelihood) of the saturated model as a measure of model fit.

## Discussion

Our findings suggest that differential habitat use was more important than spatial partitioning in structuring the Adirondack carnivore community, with relatively broad-scale shifts in space use only observed for a few species. Given the widespread observation of strong interference competition among Carnivora, often reaching extremes of intraguild predation, one might assume that spatial partitioning (one measure of intraguild competition) is an order-wide phenomenon that plays a central role in structuring carnivore communities. Unlike many other studies we took a community-wide perspective and assessed co-occurrence in some cases that were between pairs of species that are closely related and thought to strongly compete (i.e., the Mustelidae), but in other cases were not. In almost all cases, we found that distributions of taxa were best predicted by measures of habitat variation alone rather than by models that also included patterns of co-occurrence of larger or potentially competing carnivore taxa. For example, fisher occupancy was most strongly predicted by average size of natural fragments, house density, and paved road density, regardless of the presence of other carnivore species. Thus, paired interactions among the six most abundant members of the carnivore community in the Adirondacks can be viewed as less important in influencing distributions of individual species than the effects of habitat selection based on natural features and human disturbances. Of the eighteen possible interactions of taxa (seventeen of which could be modeled using an information-theoretic approach), only four were improved by adding interactions with other taxa, and of these four, only one (*C*. *latrans—Mustela spp*.) improved model fit by >2 AIC units (Fig 2).

Although they have not received the attention of studies showing strong intraguild interactions among pairs of carnivore species, a number of studies have failed to identify strong evidence of altered demography or habitat use among co-occurring carnivore species [25, 35–37]. Given the ecological diversity of the Carnivora, including foraging ecologies that range from frugivorous to strictly carnivorous, this should not be surprising as the selective basis for strong and potentially risky interference competition is in many cases unclear. Indeed, our results raise the possibility that cases lacking evidence of relatively broad-scale spatial partitioning among members of a carnivore community might be the norm rather than the exception.

As might be expected for taxa with diverse life histories and a wide weight range (<1 kg to >>100 kg), habitat models reflected a varied set of measures and scales. For instance, *U*. *americanus* was best predicted by a combination of local vegetation structure, meso-scale measures of forest cover, and large-scale measures of road networks. Fishers had higher occupancy rates in areas with higher density of houses, which is dramatically different than the behavior of fishers in the Pacific Northwest where human activities have extirpated the species from all but the least-disturbed forest habitats [38]. Our findings suggest adaptive behavior by eastern fishers to exploit resources associated with some level of human-related disturbance. For all species several habitat variables repeatedly occurred in top models, including measures of snag and course woody debris abundance, and canopy height and openness, which each occurred in top models for at least two species (S5 Table). These variables also were found to be strongly predictive of *C*. *latrans* abundance in the ADK landscape [12] and occupancy in the Central Hardwoods region [25]. These consistent findings have potentially important conservation implications because one of the biggest ecological impacts of logging is the erosion of coarse woody debris and altering forest structure [39].

At larger scales, important predictors of occupancy differed quite widely among species. Almost all taxa, including *C*. *latrans* [12], had top models that were a mix of smaller and larger scale habitat measures. The exception to this was *Mustela* spp. for which all top-ranked models were primarily comprised of predictors measured at scales of 1 km or less (S5 Table). This result is perhaps unsurprising given the small body size of this genus and evidence that *Mustela* alter their behavior due to perceptions of the risk of interference competition [40]. However, these results should be treated with caution because they combine two species (which themselves may compete) and because of the low detection probability (*p* = 0.059) for this taxon. Site occupancy estimates should be treated with caution when *p* < 0.15 as it is difficult to distinguish between poorly-detected sites and those with true absence unless a larger number of sampling sites are surveyed [28–29, 41–42]. *Mustela* spp. were also the taxon (along with *M*. *americana*) for which model support sometimes increased when other carnivore taxa were included (Fig 2). The two strongest increases in model support occurred when *C*. *latrans* abundance was included in the small mustelid models. In both cases, *C*. *latrans* abundance negatively influenced small mustelid occupancy. Coyotes have been reported as important predators of small mustelids and likely influence their distribution in certain habitats [3, 43–44].

Despite the apparent importance of *C*. *latrans* for the smallest carnivores, the lack of a stronger community-wide structuring effect by *C*. *latrans*, especially on the mid-sized *M*. *pennanti* and *P*. *lotor*, was somewhat unexpected given that the species has been shown to compete strongly with an array of sympatric mid-sized Carnivora [45–48] and in some regions is an important source of mortality of *P*. *lotor* [49–50] and *M*. *pennanti* [51–52]. *Canis latrans* entered northeastern North American in the mid-1900s [21], and the species is now ubiquitous, although variable in abundance across the ADK landscape [12]. Possibly, refugia from *C*. *latrans* are limited, and the variability in density may be insufficient to drive habitat selection at the scales examined in this study. Also, rather than alter habitat selection, smaller Carnivora may avoid the negative effects of *C*. *latrans* through alternative means such as temporal shifts in activity or altered patterns of wariness, combined with short-term adjustments in space use [20,39, 53].

Despite our intensive sampling efforts with three complementary noninvasive techniques, some carnivore species (*V*. *vulpes*, *U*. *cinereoargenteus*, *L*. *rufus*) were rarely detected and hence were excluded from our modeling efforts. It is possible that interactions with the more common carnivores that we detected have stronger effects on the distribution of these species. Furthermore, it is important to recognize that our data represent a contemporary snapshot, but that interactions in the past may have been important in determining which species are common in the Adirondacks today (i.e., the ghost of competition past; [54]). For instance, there has been speculation that the arrival of *C*. *latrans* into the Adirondack region caused a decline in *L*. *rufus* due to competition [55], which itself may have previously increased following the extirpation of *C*. *lupus*, *P*. *concolor*, and *L*. *canadensis* by the early 1900s [22].

It is well documented that *C*. *latrans* can play an important role as intraguild predator resulting in reductions in population distribution and abundance of several smaller carnivores (e.g., [3, 56–57]). In these examples the observable impacts of competition occurred in more open landscapes compared to the Adirondacks. Measurable competition is rarely constant in space and time, and the underlying mechanisms may be correlated with environmental conditions, especially habitat structure [25, 58]. In ecosystems with dense vegetative structure intraguild interaction may appear less prevalent because the scale of space use adjustment by competing species may be much smaller, thus more difficult to measure without direct observations of fine-scale movements. Canids, for example, are remarkable in their ability to perceive threats and avoid direct interactions without large generalized space use adjustments [59], but such observations are difficult in closed habitat conditions or where snow pack is less prevalent.

The results of this study should not be construed to imply that spatial partitioning is not occurring between members of the Adirondack carnivore guild. Rather, at our scale of analysis, habitat features were more important drivers of the patterns of landscape occupancy of individual species. Therefore from the perspective of community structure, intraguild interactions within in the Adirondack carnivore community do not typically result in broad-scale space use adjustments. Neutral models of community assembly assume that all taxa comprising a trophic level are ecologically equal. However, in reality ecological communities are shaped by the balance of a variety of factors, some more important than others, including interspecific interactions and the biotic and abiotic features that comprise the landscape [60]. In the case of the ADK mammalian carnivore community, the latter factors appear to be more important for predicting the distribution of the individual taxa.

## Supporting Information

S1 Dataset Dataset used for analysis in this study. (XLSX)

S1 Table Habitat model variables. Variables used for *Martes americana* (MA), *Martes pennanti* (MP), *Mustela* (weasel) spp. (MS), *Procyon lotor* (PL), and *Ursus americanus* (UA) habitat models arranged under headings that represent five primary hypotheses that might explain how local and landscape habitat attributes influence carnivore occupancy. Field-measured variables (local vegetation structure) were averaged across nine stations distributed along each transect. Landscape-scale parameters were derived from GIS-based measurements using four buffer sizes: 0.5 k, 1 k, 5 k, and 10 k.
(DOCX)

S2 Table Detection probability models. Evaluation of survey covariates in the 90% confidence set related to per survey detection probability (*p*) for carnivores in the Adirondack Mountains, New York, USA. Null *p* included for each species for assessment of relative support of top model. To estimate *p* for each taxon, we held occupancy constant [ψ(.)] and fit encounter history data from surveys at 54 sites in 2000–2002 to the candidate model set.
(DOCX)

S3 Table Spatial habitat models. Habitat model selection results in the 90% confidence set for five carnivore taxa at five spatial scales (local, 0.5k, 1k, 5k, 10k) in the Adirondack Mountains, New York, USA. We fit encounter history data from surveys at 54 sites in 2000–2002 to the candidate model set at each spatial scale for each species. For all models, probability of detection (*p*) was the most parsimonious model from stage 1 of modeling process for each species (S2 Table). The null [ψ(.)] model (occupancy held constant for all sites) is included for each species at each scale to assess relative support for top model. Variable acronyms are in S1 Table.
(DOCX)

S4 Table Variable scale models. Variable scale selection results for five carnivore taxa from 4 buffer scales in the Adirondack Mountains, New York, USA. We fit encounter history data from surveys to the model set for each variable in top-ranking habitat models beyond local scale (S3 Table). *U*. *americanus* and *M*. *pennant* models had 5 and 3 parameters, respectively. *M*. *americana*, *P*. *lotor*, and *Mustela* spp. models had 4 parameters. For all models, probability of detection (*p*) was held as the most parsimonious model from stage 1 of modeling process for each species (S2 Table), whereas estimated occupancy (ψ) varied based on buffer scale. Variable acronyms are in S1 Table.
(DOCX)

S5 Table Cross-scale habitat models. Cross-scale model selection results in the 90% confidence set estimating overall carnivore habitat occupancy in the Adirondack Mountains, New York. We fit encounter history data surveys at 54 sites in 2000–2002 to the candidate model set. For all models, probability of detection (*p*) was held as the most parsimonious model from stage 1 of modeling process for each species (S2 Table). Estimated occupancy (ψ) varied based combinations of local and larger-scale landscape and vegetative characteristics. Variable acronyms are in S1 Table.
(DOCX)

S6 Table Coefficient estimates. Coefficient estimates (±SE) for variables by species based on cross-scale model selection results in the 90% confidence set estimating overall carnivore habitat occupancy in the Adirondack Mountains, New York. We fit encounter history data surveys at 54 sites in 2000–2002 to the candidate model set. Variable acronyms are in S1 Table.
(DOCX)
