# Supplementary material for: Differential Habitat Use or Intraguild Interactions: What Structures a Carnivore Community?
Source: PLoS One. 2016 Jan 5;11(1):e0146055. doi: 10.1371/journal.pone.0146055 (PMC4711579; doi:10.1371/journal.pone.0146055)
Supplement: S1 Table — Variables used for Martes americana (MA), Martes pennanti (MP), Mustela (weasel) spp. (MS), Procyon lotor (PL), and Ursus americanus (UA) habitat models arranged under headings that represent five primary hypotheses that might explain how local and landscape habitat attributes influence carnivore occupancy. Field-measured variables (local vegetation structure) were averaged across nine stations distributed along each transect. Landscape-scale parameters were derived from GIS-based measurements using four buffer sizes: 0.5 k, 1 k, 5 k, and 10 k. (DOCX) [file pone.0146055.s002.docx]

**Supporting Information**

**S1 Table. Habitat model variables.** Variables used for *Martes americana* (MA), *Martes pennanti* (MP)*, Mustela* (weasel) spp. (MS)*, Procyon lotor* (PL), and *Ursus americanus* (UA) habitat models arranged under headings that represent five primary hypotheses that might explain how local and landscape habitat attributes influence carnivore occupancy. Field-measured variables (local vegetation structure) were averaged across nine stations distributed along each transect. Landscape-scale parameters were derived from GIS-based measurements using four buffer sizes: 0.5 k, 1 k, 5 k, and 10 k.

| **Hypothesis and**  **associated variables** | **Description** | **Source or method** | **Species models** |
| --- | --- | --- | --- |
| **Local vegetation structure** | | | |
| HEIGHT | Mean height of trees ≥ 10 cm dbh | Field measurement | MA, PL, UA |
| VOLCWD | Mean volume of course woody debris | Field measurement | MA, MP, MS, PL |
| CANOPEN | Mean canopy openness | Field measurement | MA, MP, MS, UA |
| BASNAG | Mean basal area of snags | Field measurement | MA, MP, MS, PL, UA |
| PROPSW | Proportion of coniferous trees | Field measurement | MA, MP, MS |
| **Landcover: natural edges** | | | |
| SHORE | Shoreline density for lakes, rivers and streams | GIS (NYS 1:24k Hydrography Network Coverage) | PL, UA |
| WATER | Distance to nearest water | GIS (NY-GAP Land Cover Map 8.6) | PL |
| WETLAND | Proportion of area in wetland | GIS (NY-GAP Land Cover Map 8.6) | PL, UA |
| NATFRAG | Average size of natural fragment as delimited by roads, agriculture or human developments | GIS (NY-GAP Land Cover Map 8.6) | MA, MP, MS, PL, UA |
| **Landcover: forest type** | | | |
| FORCOV | Proportion of area in forest cover | GIS (NY-GAP Land Cover Map 8.6) | MA, MP, MS, PL, UA |
| CON | Proportion of area in conifer forest | GIS (NY-GAP Land Cover Map 8.6) | MA |
| DEC | Proportion of area in deciduous forest | GIS (NY-GAP Land Cover Map 8.6) | MA, MS, UA |
| **Anthropogenic** | | | |
| dtLOGRD | Distance to nearest logging road | GIS (digitized from USGS 100k maps as compiled from USGS 1:24k topographic maps dated 1945-1980) | UA |
| dtPAVED | Distance to nearest paved road | GIS (NYS ALIS roads layer) | UA |
| dtHOUSE | Distance to nearest house | GIS (NYS Office for Real Property Services - year 2000) | PL |
| HOUSE | House density | GIS (NYS Office for Real Property Services - year 2000) | MP, PL, UA |
| PAVED | Density of paved county roads | GIS (NYS ALIS roads layer) | MA, MP, UA |
| LOGRD | Logging road density | GIS (digitized from USGS 100k maps as compiled from USGS 1:24k topographic maps dated 1945-1980) | UA |
| **Physical** | | | |
| ASPECT | Degree to which the slope was south facing | Derived from digital elevation model, NYS Dept. Environmental Conservation | PL, UA |
| ELE | Elevation | Digital elevation model, NYS Dept. Environmental Conservation | MA, MS, PL |
| TRI | Terrain ruggedness index | Digital elevation model | MA, MS, PL, UA |
| SNOW | Snowfall average winters 2002/3 and 2003/4. | GIS National (Operational Hydrologic Remote Sensing Center) | MA, MS, PL |
