# Supplementary material for: Differential Habitat Use or Intraguild Interactions: What Structures a Carnivore Community?
Source: PLoS One. 2016 Jan 5;11(1):e0146055. doi: 10.1371/journal.pone.0146055 (PMC4711579; doi:10.1371/journal.pone.0146055)
Supplement: S2 Table — Evaluation of survey covariates in the 90% confidence set related to per survey detection probability (p) for carnivores in the Adirondack Mountains, New York, USA. Null p included for each species for assessment of relative support of top model. To estimate p for each taxon, we held occupancy constant [ψ(.)] and fit encounter history data from surveys at 54 sites in 2000–2002 to the candidate model set. (DOCX) [file pone.0146055.s003.docx]

**Supporting Information**

**S2 Table. Detection probability models.** Evaluation of survey covariates in the 90% confidence set related to per survey detection probability (*p*) for carnivores in the Adirondack Mountains, New York, USA. Null *p* included for each species for assessment of relative support of top model. To estimate *p* for each taxon, we held occupancy constant [ψ(.)] and fit encounter history data from surveys at 54 sites in 2000–2002 to the candidate model set.

| **Model** | **AIC_c_**^a^ | **ΔAIC_c_** | ***w***^b^ | **K**^c^ | **Deviance**^d^ |
| --- | --- | --- | --- | --- | --- |
| *U. americanus* | | | | | |
| *p*(1-3,4,5^e^) | 260.61 | 0.00 | 0.876 | 4 | 251.79 |
| *p*(INT^f^ ) | 265.58 | 4.97 | 0.073 | 6 | 251.79 |
| *p*(.^g^) | 268.02 | 7.41 | 0.021 | 2 | 263.78 |
| *M. pennanti* | | | | | |
| *p*(PREVDET^h^ ) | 346.47 | 0.00 | 0.690 | 9 | 328.47 |
| *p*(METHOD^i^ +PREVDET) | 348.07 | 1.60 | 0.310 | 10 | 328.07 |
| *p*(.) | 400.35 | 53.88 | 0.000 | 2 | 396.35 |
| *M. americana* | | | | | |
| *p*(METHOD) | 128.77 | 0 | 0.807 | 3 | 122.77 |
| *p*(INT) | 132.25 | 3.48 | 0.142 | 10 | 112.25 |
| *p*(.) | 137.26 | 8.49 | 0.011 | 2 | 133.26 |
| *P. lotor* | | | | | |
| *p*(METHOD) | 316.65 | 0.00 | 0.564 | 3 | 310.17 |
| *p*(.) | 317.34 | 0.69 | 0.399 | 2 | 313.10 |
| *Mustela* (weasel) spp. | | | | | |
| *p*(METHOD) | 101.83 | 0.00 | 0.774 | 3 | 95.83 |
| *p*(.) | 105.43 | 3.60 | 0.128 | 2 | 101.43 |

^a^ Akaike Information Criterion for small samples

^b^ Model probability

^c^ Number of model parameters

^d^ Difference in -2Log(Likelihood) of the current model and -2log(Likelihood) of the saturated

model as a measure of model fit

^e^ Surveys 1–3 common intercept; surveys 4 and 5 independent intercept

^f^ Independent intercept for each survey

^g^ Null model; all surveys held to common intercept and slope

^h^ Previous detection at a site during a previous survey

^i^ Method used to detect species (remote camera vs. track-plate)
