# Supplementary material for: Differential Habitat Use or Intraguild Interactions: What Structures a Carnivore Community?
Source: PLoS One. 2016 Jan 5;11(1):e0146055. doi: 10.1371/journal.pone.0146055 (PMC4711579; doi:10.1371/journal.pone.0146055)
Supplement: S3 Table — Habitat model selection results in the 90% confidence set for five carnivore taxa at five spatial scales (local, 0.5k, 1k, 5k, 10k) in the Adirondack Mountains, New York, USA. We fit encounter history data from surveys at 54 sites in 2000–2002 to the candidate model set at each spatial scale for each species. For all models, probability of detection (p) was the most parsimonious model from stage 1 of modeling process for each species (S2 Table). The null [ψ(.)] model (occupancy held constant for all sites) is included for each species at each scale to assess relative support for top model. Variable acronyms are in S1 Table. (DOCX) [file pone.0146055.s004.docx]

**Supporting Information**

**S3 Table. Spatial habitat models.** Habitat model selection results in the 90% confidence set for five carnivore taxa at five spatial scales (local, 0.5k, 1k, 5k, 10k) in the Adirondack Mountains, New York, USA. We fit encounter history data from surveys at 54 sites in 2000–2002 to the candidate model set at each spatial scale for each species. For all models, probability of detection (*p*) was the most parsimonious model from stage 1 of modeling process for each species (S2 Table). The null [ψ(.)] model (occupancy held constant for all sites) is included for each species at each scale to assess relative support for top model. Variable acronyms are in S1 Table.

| **Model** | **AIC_c_**^a^ | **ΔAIC_c_** | ***w***^b^ | **K**^c^ | **Deviance**^d^ |
| --- | --- | --- | --- | --- | --- |
| *U. americanus* local | | | | | |
| ψ(BASNAG) | 254.00 | 0.00 | 0.353 | 5 | 242.75 |
| ψ(BASNAG + dtLOGRD) | 255.39 | 1.39 | 0.176 | 6 | 241.60 |
| ψ(BASNAG + HEIGHT) | 256.04 | 2.04 | 0.127 | 6 | 242.25 |
| ψ(BASNAG + CANOPEN) | 256.06 | 2.06 | 0.126 | 6 | 242.27 |
| ψ(BASNAG + dtPAVED) | 256.54 | 2.54 | 0.099 | 6 | 242.75 |
| ψ(BASNAG + HEIGHT + CANOPEN) | 257.32 | 3.32 | 0.067 | 7 | 240.89 |
| ψ(.) | 260.61 | 6.61 | 0.013 | 4 | 251.79 |
| *U. americanus* 0.5k | | | | | |
| ψ(FORCOV + LOGRD) | 256.63 | 0.00 | 0.137 | 6 | 242.84 |
| ψ(FORCOV + PAVED + LOGRD) | 256.70 | 0.07 | 0.132 | 7 | 240.27 |
| ψ(LOGRD + NATFRAG) | 256.96 | 0.33 | 0.116 | 6 | 243.17 |
| ψ(PAVED + LOGRD + NATFRAG) | 258.36 | 1.73 | 0.058 | 7 | 241.93 |
| ψ(PAVED + LOGRD) | 258.46 | 1.83 | 0.055 | 6 | 244.67 |
| ψ(PAVED) | 259.04 | 2.41 | 0.041 | 5 | 247.79 |
| ψ(NATFRAG) | 259.21 | 2.58 | 0.038 | 5 | 247.96 |
| ψ(HOUSE + LOGRD) | 259.25 | 2.62 | 0.037 | 6 | 245.46 |
| ψ(LOGRD) | 259.34 | 2.71 | 0.035 | 5 | 248.09 |
| ψ(FORCOV + PAVED + LOGRD + HOUSE) | 259.38 | 2.75 | 0.035 | 8 | 240.18 |
| ψ(FORCOV + PAVED) | 259.46 | 2.83 | 0.033 | 6 | 245.67 |
| ψ(TRI + LOGRD) | 259.92 | 3.29 | 0.026 | 6 | 246.13 |
| ψ(PAVED + NATFRAG) | 259.99 | 3.36 | 0.026 | 6 | 246.20 |
| ψ(HOUSE) | 260.00 | 3.37 | 0.025 | 5 | 248.75 |
| ψ(HOUSE + NATFRAG) | 260.20 | 3.57 | 0.023 | 6 | 246.41 |
| ψ(FORCOV + NATFRAG) | 260.57 | 3.94 | 0.019 | 6 | 246.78 |
| ψ(.) | 260.61 | 3.98 | 0.019 | 4 | 251.79 |
| ψ(FORCOV + DEC) | 260.84 | 4.21 | 0.017 | 6 | 247.05 |
| ψ(HOUSE + PAVED + LOGRD) | 260.88 | 4.25 | 0.016 | 7 | 244.45 |
| ψ(FORCOV) | 261.02 | 4.39 | 0.015 | 5 | 249.77 |
| *U. americanus* 1k | | | | | |
| ψ(LOGRD + NATFRAG) | 255.87 | 0.00 | 0.189 | 6 | 242.08 |
| ψ(PAVED + LOGRD + NATFRAG) | 257.10 | 1.23 | 0.102 | 7 | 240.67 |
| ψ(FORCOV + LOGRD) | 257.64 | 1.77 | 0.078 | 6 | 243.85 |
| ψ(PAVED + LOGRD) | 257.75 | 1.88 | 0.074 | 6 | 243.96 |
| ψ(FORCOV + PAVED + LOGRD) | 257.84 | 1.97 | 0.071 | 7 | 241.41 |
| ψ(HOUSE + LOGRD + NATFRAG) | 258.38 | 2.51 | 0.054 | 7 | 241.95 |
| ψ(PAVED) | 258.80 | 2.93 | 0.044 | 5 | 247.55 |
| ψ(LOGRD) | 258.94 | 3.07 | 0.041 | 5 | 247.69 |
| ψ(NATFRAG) | 258.95 | 3.08 | 0.040 | 5 | 247.70 |
| ψ(HOUSE + PAVED + LOGRDk + NATFRAG) | 259.38 | 3.51 | 0.033 | 8 | 240.18 |
| ψ(LOGRD + TRI) | 259.52 | 3.65 | 0.030 | 6 | 245.73 |
| ψ(PAVED + NATFRAG) | 259.90 | 4.03 | 0.025 | 6 | 246.11 |
| ψ(HOUSE + LOGRD) | 260.04 | 4.17 | 0.024 | 6 | 246.25 |
| ψ(HOUSE + PAVED + LOGRD) | 260.32 | 4.45 | 0.020 | 7 | 243.89 |
| ψ(FORCOV + PAVED + LOGRD + HOUSE) | 260.58 | 4.71 | 0.018 | 8 | 241.38 |
| ψ(.) | 260.61 | 4.74 | 0.018 | 4 | 251.79 |
| ψ(FORCOV + PAVED) | 260.94 | 5.07 | 0.015 | 6 | 247.15 |
| ψ(HOUSE + NATFRAG) | 261.12 | 5.25 | 0.014 | 6 | 247.33 |
| ψ(FORCOV + NATFRAG) | 261.13 | 5.26 | 0.014 | 6 | 247.34 |
| *U. americanus* 5k | | | | | |
| ψ(LOGRD + NATFRAG) | 257.74 | 0.00 | 0.124 | 6 | 243.95 |
| ψ(FORCOV + DEC) | 257.97 | 0.23 | 0.111 | 6 | 244.18 |
| ψ(NATFRAG) | 258.10 | 0.36 | 0.104 | 5 | 246.85 |
| ψ(PAVED) | 258.12 | 0.38 | 0.103 | 5 | 246.87 |
| ψ(PAVED + LOGRD) | 259.24 | 1.50 | 0.059 | 6 | 245.45 |
| ψ(PAVED + NATFRAG) | 259.50 | 1.76 | 0.052 | 6 | 245.71 |
| ψ(PAVED + LOGRD + NATFRAG) | 259.59 | 1.85 | 0.049 | 7 | 243.16 |
| ψ(HOUSE + PAVED) | 259.63 | 1.89 | 0.048 | 6 | 245.84 |
| ψ(HOUSE + PAVED + LOGRD) | 260.19 | 2.45 | 0.037 | 7 | 243.76 |
| ψ(HOUSE + LOGRD + NATFRAG) | 260.34 | 2.60 | 0.034 | 7 | 243.91 |
| ψ(TRI + NATFRAG) | 260.44 | 2.70 | 0.032 | 6 | 246.65 |
| ψ(FORCOV + NATFRAG) | 260.59 | 2.85 | 0.030 | 6 | 246.80 |
| ψ(.) | 260.61 | 2.87 | 0.030 | 4 | 251.79 |
| ψ(FORCOV + PAVED) | 260.66 | 2.92 | 0.029 | 6 | 246.87 |
| ψ(HOUSE + PAVED + LOGRD + NATFRAG) | 261.60 | 3.86 | 0.018 | 8 | 242.40 |
| ψ(HOUSE + PAVED + NATFRAG) | 261.66 | 3.92 | 0.018 | 7 | 245.23 |
| ψ(TRI) | 261.66 | 3.92 | 0.018 | 5 | 250.41 |
| ψ(HOUSE) | 261.69 | 3.95 | 0.017 | 5 | 250.44 |
| *U. americanus* 10k | | | | | |
| ψ(LOGRD + NATFRAG) | 253.38 | 0.00 | 0.182 | 6 | 239.59 |
| ψ(HOUSE + PAVED + LOGRD) | 253.52 | 0.14 | 0.170 | 7 | 237.09 |
| ψ(PAVED + LOGRD) | 254.31 | 0.93 | 0.114 | 6 | 240.52 |
| ψ(PAVED) | 255.39 | 2.01 | 0.067 | 5 | 244.14 |
| ψ(HOUSE + PAVED) | 255.66 | 2.28 | 0.058 | 6 | 241.87 |
| ψ(HOUSE + PAVED + LOGRD + NATFRAG) | 255.82 | 2.44 | 0.054 | 8 | 236.62 |
| ψ(HOUSE + LOGRD + NATFRAG) | 256.01 | 2.63 | 0.049 | 7 | 239.58 |
| ψ(HOUSE + PAVED + LOGRD + FORCOV) | 256.27 | 2.89 | 0.043 | 8 | 237.07 |
| ψ(LOGRD + TRI) | 256.66 | 3.28 | 0.035 | 6 | 242.87 |
| ψ(FORCOV + PAVED + LOGRD) | 256.83 | 3.45 | 0.032 | 7 | 240.40 |
| ψ(NATFRAG) | 257.13 | 3.75 | 0.028 | 5 | 245.88 |
| ψ(PAVED + NATFRAG) | 257.28 | 3.90 | 0.026 | 6 | 243.49 |
| ψ(FORCOV + PAVED) | 257.93 | 4.55 | 0.019 | 6 | 244.14 |
| ψ(HOUSE + PAVED + FORCOV) | 258.17 | 4.79 | 0.017 | 7 | 241.74 |
| ψ(HOUSE + PAVED + NATFRAG) | 258.29 | 4.91 | 0.016 | 7 | 241.86 |
| ψ(.) | 260.61 | 7.23 | 0.005 | 4 | 251.79 |
| *M. pennanti* local | | | | | |
| ψ(.) | 400.59 | 0.00 | 0.176 | 2 | 396.35 |
| ψ(BASNAG) | 400.90 | 0.31 | 0.151 | 3 | 394.42 |
| ψ(CANOPEN) | 401.19 | 0.60 | 0.130 | 3 | 394.71 |
| ψ(VOLCWD) | 401.31 | 0.72 | 0.123 | 3 | 394.83 |
| ψ(CANOPEN + BASNAG) | 402.14 | 1.55 | 0.081 | 4 | 393.32 |
| ψ(PROPSW) | 402.69 | 2.10 | 0.062 | 3 | 396.21 |
| ψ(CANOPEN + VOLCWD) | 402.76 | 2.17 | 0.060 | 4 | 393.94 |
| ψ(BASNAG + VOLCWD) | 403.00 | 2.41 | 0.053 | 4 | 394.18 |
| ψ(BASNAG + PROPSW) | 403.16 | 2.57 | 0.049 | 4 | 394.34 |
| ψ(CANOPEN + PROPSW) | 403.19 | 2.60 | 0.048 | 4 | 394.37 |
| ψ(VOLCWD + PROPSW) | 403.37 | 2.78 | 0.044 | 4 | 394.55 |
| *M. pennanti* 0.5k | | | | | |
| ψ(NATFRAG) | 399.61 | 0.00 | 0.233 | 3 | 393.13 |
| ψ(FORCOV + NATFRAG) | 400.39 | 0.78 | 0.158 | 4 | 391.57 |
| ψ(.) | 400.59 | 0.98 | 0.143 | 2 | 396.35 |
| ψ(PAVED + NATFRAG) | 401.08 | 1.47 | 0.112 | 4 | 392.26 |
| ψ(HOUSE + NATFRAG) | 401.94 | 2.33 | 0.073 | 4 | 393.12 |
| ψ(FORCOV) | 402.06 | 2.45 | 0.068 | 3 | 395.58 |
| ψ(HOUSE) | 402.53 | 2.92 | 0.054 | 3 | 396.05 |
| ψ(PAVED) | 402.83 | 3.22 | 0.047 | 3 | 396.35 |
| ψ(PAVED + NATFRAG + HOUSE) | 402.91 | 3.30 | 0.045 | 5 | 391.66 |
| *M. pennanti* 1k | | | | | |
| ψ(PAVED + NATFRAG + HOUSE) | 394.81 | 0.00 | 0.546 | 5 | 383.56 |
| ψ(PAVED + HOUSE) | 398.16 | 3.35 | 0.102 | 4 | 389.34 |
| ψ(NATFRAG + HOUSE) | 398.79 | 3.98 | 0.075 | 4 | 389.97 |
| ψ(HOUSE) | 398.79 | 3.98 | 0.075 | 3 | 392.31 |
| ψ(NATFRAG) | 399.25 | 4.44 | 0.059 | 3 | 392.77 |
| ψ(PAVED + NATFRAG) | 399.94 | 5.13 | 0.042 | 4 | 391.12 |
| ψ(.) | 400.59 | 5.78 | 0.030 | 2 | 396.35 |
| *M. pennanti* 5k | | | | | |
| ψ(PAVED + NATFRAG + HOUSE) | 394.43 | 0.00 | 0.391 | 5 | 383.18 |
| ψ(HOUSE) | 395.90 | 1.47 | 0.188 | 3 | 389.42 |
| ψ(NATFRAG + HOUSE) | 396.00 | 1.57 | 0.178 | 4 | 387.18 |
| ψ(PAVED + HOUSE) | 397.85 | 3.42 | 0.071 | 4 | 389.03 |
| ψ(FORCOV + HOUSE) | 398.24 | 3.81 | 0.058 | 4 | 389.42 |
| ψ(NATFRAG) | 398.98 | 4.55 | 0.040 | 3 | 392.50 |
| ψ(.) | 400.59 | 6.16 | 0.018 | 2 | 396.35 |
| *M. pennanti* 10k | | | | | |
| ψ(NATFRAG) | 398.75 | 0.00 | 0.246 | 3 | 392.27 |
| ψ(PAVED) | 400.40 | 1.65 | 0.108 | 3 | 393.92 |
| ψ(HOUSE + NATFRAG) | 400.53 | 1.78 | 0.101 | 4 | 391.71 |
| ψ(HOUSE) | 400.59 | 1.84 | 0.098 | 3 | 394.11 |
| ψ(.) | 400.59 | 1.84 | 0.098 | 2 | 396.35 |
| ψ(FORCOV + NATFRAG) | 400.82 | 2.07 | 0.088 | 4 | 392.00 |
| ψ(PAVED + NATFRAG) | 400.96 | 2.21 | 0.082 | 4 | 392.14 |
| ψ(PAVED + FORCOV) | 402.27 | 3.52 | 0.042 | 4 | 393.45 |
| ψ(HOUSE + PAVED) | 402.58 | 3.83 | 0.036 | 4 | 393.76 |
| ψ(PAVED + NATFRAG + HOUSE) | 402.59 | 3.84 | 0.036 | 5 | 391.34 |
| *M. americana* local | | | | | |
| ψ(CANOPEN + BASNAG + HEIGHT) | 121.59 | 0.00 | 0.330 | 6 | 107.80 |
| ψ(BASNAG + CANOPEN) | 122.48 | 0.89 | 0.212 | 5 | 111.23 |
| ψ(CANOPEN + BASNAG + HEIGHT + VOLCWD) | 123.58 | 1.99 | 0.122 | 7 | 107.15 |
| ψ(CANOPEN) | 125.53 | 3.94 | 0.046 | 4 | 116.71 |
| ψ(VOLCWD + CANOPEN) | 125.63 | 4.04 | 0.044 | 5 | 114.38 |
| ψ(BASNAG) | 125.65 | 4.06 | 0.043 | 4 | 116.83 |
| ψ(HEIGHT + CANOPEN) | 125.99 | 4.40 | 0.037 | 5 | 114.74 |
| ψ(BASNAG + HEIGHT) | 126.22 | 4.63 | 0.033 | 5 | 114.97 |
| ψ(PROPSW + BASNAG) | 126.51 | 4.92 | 0.028 | 5 | 115.26 |
| ψ(VOLCWD + BASNAG + HEIGHT) | 127.10 | 5.51 | 0.021 | 6 | 113.31 |
| ψ(.) | 129.25 | 7.66 | 0.007 | 3 | 122.77 |
| *M. americana* 0.5k | | | | | |
| ψ(TRI) | 119.86 | 0.00 | 0.443 | 4 | 111.05 |
| ψ(ELE + TRI) | 121.46 | 1.60 | 0.199 | 5 | 110.21 |
| ψ(FORCOV + TRI) | 122.30 | 2.44 | 0.131 | 5 | 111.05 |
| ψ(ELE) | 122.66 | 2.80 | 0.109 | 4 | 113.85 |
| ψ(SNOW + ELE) | 125.09 | 5.23 | 0.032 | 5 | 113.84 |
| ψ(.) | 129.25 | 9.39 | 0.009 | 3 | 122.77 |
| *M. americana* 1k | | | | | |
| ψ(TRI) | 120.18 | 0.00 | 0.323 | 4 | 111.36 |
| ψ(ELE + TRI) | 121.74 | 1.56 | 0.148 | 5 | 110.49 |
| ψ(ELE) | 122.11 | 1.93 | 0.123 | 4 | 113.30 |
| ψ(SNOW + CON + TRI) | 122.37 | 2.19 | 0.108 | 6 | 108.58 |
| ψ(FORCOV + TRI) | 122.55 | 2.37 | 0.099 | 5 | 111.30 |
| ψ(FORCOV + NATFRAG) | 123.69 | 3.51 | 0.056 | 5 | 112.44 |
| ψ(SNOW + ELE) | 124.54 | 4.36 | 0.037 | 5 | 113.29 |
| ψ(FORCOV + PAVED) | 124.94 | 4.76 | 0.030 | 5 | 113.69 |
| ψ(.) | 129.25 | 9.07 | 0.010 | 3 | 122.77 |
| *M. americana* 5k | | | | | |
| ψ(TRI) | 117.12 | 0.00 | 0.250 | 4 | 108.31 |
| ψ(ELE) | 118.15 | 1.03 | 0.150 | 4 | 109.33 |
| ψ(FORCOV + NATFRAG) | 118.41 | 1.29 | 0.131 | 5 | 107.16 |
| ψ(ELE + TRI) | 118.58 | 1.46 | 0.121 | 5 | 107.33 |
| ψ(SNOW + TRI) | 118.73 | 1.61 | 0.112 | 5 | 107.48 |
| ψ(FORCOV + TRI) | 119.25 | 2.13 | 0.086 | 5 | 108.00 |
| ψ(ELE + SNOW) | 120.58 | 3.46 | 0.044 | 5 | 109.33 |
| ψ(SNOW + CON + TRI) | 121.08 | 3.96 | 0.035 | 6 | 107.29 |
| ψ(.) | 129.25 | 12.13 | 0.002 | 3 | 122.77 |
| *M. americana* 10k | | | | | |
| ψ(TRI) | 114.67 | 0.00 | 0.292 | 4 | 105.86 |
| ψ(ELE + TRI) | 115.27 | 0.60 | 0.216 | 5 | 104.02 |
| ψ(ELE) | 115.91 | 1.24 | 0.157 | 4 | 107.09 |
| ψ(FORCOV + TRI) | 117.09 | 2.42 | 0.087 | 5 | 105.84 |
| ψ(SNOW + TRI) | 117.10 | 2.43 | 0.087 | 5 | 105.85 |
| ψ(SNOW + ELE) | 118.25 | 3.58 | 0.049 | 5 | 107.00 |
| ψ(FORCOV + NATFRAG) | 118.75 | 4.08 | 0.038 | 5 | 107.50 |
| ψ(.) | 129.25 | 14.58 | 0.000 | 3 | 122.77 |
| *P. lotor* local | | | | | |
| ψ(.) | 316.65 | 0.00 | 0.273 | 3 | 310.17 |
| ψ(BASNAG) | 317.78 | 1.13 | 0.155 | 4 | 308.96 |
| ψ(HEIGHT) | 318.16 | 1.51 | 0.128 | 4 | 309.34 |
| ψ(dtHOUSE) | 318.80 | 2.15 | 0.093 | 4 | 309.98 |
| ψ(WATER) | 318.97 | 2.32 | 0.086 | 4 | 310.15 |
| ψ(VOLCWD) | 318.97 | 2.32 | 0.086 | 4 | 310.15 |
| ψ(VOLCWD + BASNAG) | 319.77 | 3.12 | 0.057 | 5 | 308.52 |
| ψ(BASNAG + HEIGHT) | 319.81 | 3.16 | 0.056 | 5 | 308.56 |
| *P. lotor* 0.5k | | | | | |
| ψ(TRI) | 315.47 | 0.00 | 0.255 | 4 | 306.65 |
| ψ(TRI + ASPECT) | 316.16 | 0.69 | 0.180 | 5 | 304.91 |
| ψ(.) | 316.65 | 1.18 | 0.141 | 3 | 310.17 |
| Ψ(ASPECT) | 317.35 | 1.88 | 0.099 | 4 | 308.53 |
| ψ(HOUSE) | 317.65 | 2.18 | 0.086 | 4 | 308.83 |
| ψ(NATFRAG) | 318.60 | 3.13 | 0.053 | 4 | 309.78 |
| ψ(SHORE) | 318.65 | 3.18 | 0.052 | 4 | 309.83 |
| ψ(WETLAND) | 318.73 | 3.26 | 0.050 | 4 | 309.91 |
| *P. lotor* 1k | | | | | |
| ψ(TRI) | 315.79 | 0.00 | 0.221 | 4 | 306.97 |
| ψ(.) | 316.65 | 0.86 | 0.144 | 3 | 310.17 |
| ψ(TRI + HOUSE) | 316.84 | 1.05 | 0.131 | 5 | 305.59 |
| ψ(HOUSE) | 316.91 | 1.12 | 0.126 | 4 | 308.09 |
| ψ(TRI + NATFRAG) | 318.22 | 2.43 | 0.066 | 5 | 306.97 |
| ψ(NATFRAG) | 318.48 | 2.69 | 0.058 | 4 | 309.66 |
| ψ(WETLAND) | 318.82 | 3.03 | 0.049 | 4 | 310.00 |
| ψ(FORCOV) | 318.98 | 3.19 | 0.045 | 4 | 310.16 |
| ψ(WETLAND + SHORE) | 319.14 | 3.35 | 0.041 | 5 | 307.89 |
| ψ(NATFRAG + HOUSE) | 319.29 | 3.50 | 0.038 | 5 | 308.04 |
| *P. lotor* 5k | | | | | |
| ψ(.) | 316.65 | 0.00 | 0.140 | 3 | 310.17 |
| ψ(HOUSE) | 316.69 | 0.04 | 0.137 | 4 | 307.87 |
| ψ(TRI) | 316.72 | 0.07 | 0.135 | 4 | 307.90 |
| ψ(SHORE) | 317.36 | 0.71 | 0.098 | 4 | 308.54 |
| ψ(TRI + HOUSE) | 317.81 | 1.16 | 0.079 | 5 | 306.56 |
| ψ(SNOW) | 317.90 | 1.25 | 0.075 | 4 | 309.08 |
| ψ(ELE) | 318.02 | 1.37 | 0.071 | 4 | 309.20 |
| ψ(WETLAND) | 318.59 | 1.94 | 0.053 | 4 | 309.77 |
| ψ(FORCOV) | 318.98 | 2.33 | 0.044 | 4 | 310.16 |
| ψ(TRI + NATFRAG) | 319.11 | 2.46 | 0.041 | 5 | 307.86 |
| ψ(NATFRAG + HOUSE) | 319.12 | 2.47 | 0.041 | 5 | 307.87 |
| *P. lotor* 10k | | | | | |
| ψ(.) | 316.65 | 0 | 0.147 | 3 | 310.17 |
| ψ(TRI) | 316.71 | 0.06 | 0.1426 | 4 | 307.89 |
| ψ(SNOW) | 317.55 | 0.9 | 0.0937 | 4 | 308.73 |
| ψ(TRI + HOUSE) | 317.72 | 1.07 | 0.0861 | 5 | 306.47 |
| ψ(WETLAND) | 317.92 | 1.27 | 0.0779 | 4 | 309.1 |
| ψ(HOUSE) | 317.99 | 1.34 | 0.0752 | 4 | 309.17 |
| ψ(ELE) | 318.22 | 1.57 | 0.067 | 4 | 309.4 |
| ψ(SHORE) | 318.25 | 1.6 | 0.066 | 4 | 309.43 |
| ψ(NATFRAG + HOUSE) | 318.52 | 1.87 | 0.0577 | 5 | 307.27 |
| ψ(FORCOV) | 318.89 | 2.24 | 0.048 | 4 | 310.07 |
| ψ(TRI + NATFRAG) | 319.11 | 2.46 | 0.043 | 5 | 307.86 |
| *Mustela* local | | | | | |
| ψ(PROPSW) | 101.41 | 0.00 | 0.283 | 4 | 92.59 |
| ψ(.) | 102.31 | 0.90 | 0.180 | 3 | 95.83 |
| ψ(BASNAG) | 102.77 | 1.36 | 0.143 | 4 | 93.95 |
| ψ(PROPSW + VOLCWD) | 103.73 | 2.32 | 0.089 | 5 | 92.48 |
| ψ(PROPSW + CANOPEN) | 103.79 | 2.38 | 0.086 | 5 | 92.54 |
| ψ(CANOPEN) | 104.33 | 2.92 | 0.066 | 4 | 95.51 |
| ψ(VOLCWD) | 104.43 | 3.02 | 0.063 | 4 | 95.61 |
| *Mustela* 0.5k | | | | | |
| ψ(FORCOV) | 96.21 | 0.00 | 0.355 | 4 | 87.39 |
| ψ(DEC) | 97.60 | 1.39 | 0.177 | 4 | 88.78 |
| ψ(TRI + FORCOV) | 98.10 | 1.89 | 0.138 | 5 | 86.85 |
| ψ(ELE + FORCOV) | 98.72 | 2.51 | 0.101 | 5 | 87.47 |
| ψ(TRI + DEC) | 99.16 | 2.95 | 0.081 | 5 | 87.91 |
| ψ(ELE + DEC) | 100.02 | 3.81 | 0.053 | 5 | 88.77 |
| ψ(.) | 102.31 | 6.1 | 0.017 | 3 | 95.83 |
| *Mustela* 1k | | | | | |
| ψ(DEC) | 92.35 | 0.00 | 0.575 | 4 | 83.53 |
| ψ(SNOW + NATFRAG) | 95.12 | 2.77 | 0.144 | 5 | 83.87 |
| ψ(ELE + FORCOV) | 95.85 | 3.50 | 0.100 | 5 | 84.60 |
| ψ(ELE + NATFRAG) | 95.85 | 3.50 | 0.100 | 5 | 84.60 |
| ψ(.) | 102.31 | 9.96 | 0.004 | 3 | 95.83 |
| *Mustela* 5k | | | | | |
| ψ(ELE + DEC) | 98.11 | 0.00 | 0.274 | 5 | 86.86 |
| ψ(TRI + ELE) | 98.50 | 0.39 | 0.225 | 5 | 87.25 |
| ψ(SNOW + TRI) | 99.52 | 1.41 | 0.135 | 5 | 88.27 |
| ψ(DEC) | 99.66 | 1.55 | 0.126 | 4 | 90.85 |
| ψ(TRI) | 101.39 | 3.28 | 0.053 | 4 | 92.57 |
| ψ(SNOW) | 101.55 | 3.44 | 0.049 | 4 | 92.73 |
| ψ(NATFRAG) | 101.95 | 3.84 | 0.040 | 4 | 93.13 |
| ψ(.) | 102.31 | 4.20 | 0.034 | 3 | 95.83 |
| *Mustela* 10k | | | | | |
| ψ(FORCOV + NATFRAG) | 95.41 | 0.00 | 0.448 | 5 | 84.16 |
| ψ(SNOW) | 98.21 | 2.80 | 0.111 | 4 | 89.39 |
| ψ(SNOW + ELE) | 99.25 | 3.84 | 0.066 | 5 | 88.00 |
| ψ(FORCOV) | 99.38 | 3.97 | 0.062 | 4 | 90.56 |
| ψ(DEC) | 99.62 | 4.21 | 0.055 | 4 | 90.80 |
| ψ(ELE + NATFRAG) | 99.84 | 4.43 | 0.049 | 5 | 88.59 |
| ψ(ELE + DEC) | 100.08 | 4.67 | 0.043 | 5 | 88.83 |
| ψ(ELE + FORCOV) | 100.55 | 5.14 | 0.034 | 5 | 89.30 |
| ψ(NATFRAG) | 100.73 | 5.32 | 0.031 | 4 | 91.92 |
| ψ(TRI) | 101.21 | 5.80 | 0.025 | 4 | 92.39 |
| ψ(.) | 102.31 | 6.90 | 0.011 | 3 | 95.83 |

^a^ Akaike Information Criterion for small samples

^b^ Model probability

^c^ Number of model parameters

^d^ Difference in -2Log(Likelihood) of the current model and -2log(Likelihood) of the saturated

model as a measure of model fit
