# Supplementary material for: Differential Habitat Use or Intraguild Interactions: What Structures a Carnivore Community?
Source: PLoS One. 2016 Jan 5;11(1):e0146055. doi: 10.1371/journal.pone.0146055 (PMC4711579; doi:10.1371/journal.pone.0146055)
Supplement: S4 Table — Variable scale selection results for five carnivore taxa from 4 buffer scales in the Adirondack Mountains, New York, USA. We fit encounter history data from surveys to the model set for each variable in top-ranking habitat models beyond local scale (S3 Table). U. americanus and M. pennant models had 5 and 3 parameters, respectively. M. americana, P. lotor, and Mustela spp. models had 4 parameters. For all models, probability of detection (p) was held as the most parsimonious model from stage 1 of modeling process for each species (S2 Table), whereas estimated occupancy (ψ) varied based on buffer scale. Variable acronyms are in S1 Table. (DOCX) [file pone.0146055.s005.docx]

**Supporting Information**

**S4 Table. Variable scale models.** Variable scale selection results for five carnivore taxa from 4 buffer scales in the Adirondack Mountains, New York, USA. We fit encounter history data from surveys to the model set for each variable in top-ranking habitat models beyond local scale (S3 Table). *U. americanus* and *M. pennant* models had 5 and 3 parameters, respectively. *M. americana*, *P. lotor*, and *Mustela* spp. models had 4 parameters. For all models, probability of detection (*p*) was held as the most parsimonious model from stage 1 of modeling process for each species (S2 Table), whereas estimated occupancy (ψ) varied based on buffer scale. Variable acronyms are in S1 Table.

| **Model** | **AIC_c_**^a^ | **ΔAIC_c_** | ***w***^b^ | **Deviance**^c^ |
| --- | --- | --- | --- | --- |
| *U. americanus* DEC | | | | |
| ψ(5k) | 257.48 | 0.00 | 0.591 | 246.23 |
| ψ(10k) | 259.00 | 1.52 | 0.276 | 247.75 |
| ψ(1k) | 261.52 | 4.04 | 0.078 | 250.27 |
| ψ(0.5k) | 262.26 | 4.78 | 0.054 | 251.01 |
| *U. americanus* FORCOV | | | | |
| ψ(0.5k) | 261.02 | 0.00 | 0.371 | 249.77 |
| ψ(10k) | 261.86 | 0.84 | 0.244 | 250.61 |
| ψ(1k) | 262.12 | 1.10 | 0.214 | 250.87 |
| ψ(5k) | 262.58 | 1.56 | 0.170 | 251.33 |
| *U. americanus* HOUSE | | | | |
| ψ(0.5k) | 260.00 | 0.00 | 0.359 | 248.75 |
| ψ(10k) | 260.18 | 0.18 | 0.328 | 248.93 |
| ψ(1k) | 261.62 | 1.62 | 0.160 | 250.37 |
| ψ(5k) | 261.69 | 1.69 | 0.154 | 250.44 |
| *U. americanus* LOGRD | | | | |
| ψ(10k) | 258.46 | 0.00 | 0.382 | 247.21 |
| ψ(1k) | 258.94 | 0.48 | 0.301 | 247.69 |
| ψ(0.5k) | 259.34 | 0.88 | 0.246 | 248.09 |
| ψ(5k) | 261.81 | 3.35 | 0.072 | 250.56 |
| *U. americanus* NATFRAG | | | | |
| ψ(10k) | 257.13 | 0.00 | 0.422 | 245.88 |
| ψ(5k) | 258.10 | 0.97 | 0.260 | 246.85 |
| ψ(1k) | 258.95 | 1.82 | 0.170 | 247.70 |
| ψ(0.5k) | 259.21 | 2.08 | 0.149 | 247.96 |
| *U. americanus* PAVED | | | | |
| ψ(10k) | 255.39 | 0.00 | 0.626 | 244.14 |
| ψ(5k) | 258.12 | 2.73 | 0.160 | 246.87 |
| ψ(1k) | 258.80 | 3.41 | 0.114 | 247.55 |
| ψ(0.5k) | 259.04 | 3.65 | 0.101 | 247.79 |
| *M. pennanti* HOUSE | | | | |
| ψ(5k) | 395.90 | 0.00 | 0.731 | 389.42 |
| ψ(1k) | 398.79 | 2.89 | 0.172 | 392.31 |
| ψ(10k) | 400.59 | 4.69 | 0.070 | 394.11 |
| ψ(0.5k) | 402.53 | 6.63 | 0.027 | 396.05 |
| *M. pennanti* FORCOV | | | | |
| ψ(0.5k) | 402.06 | 0.00 | 0.324 | 395.58 |
| ψ(1k) | 402.76 | 0.70 | 0.229 | 396.28 |
| ψ(5k) | 402.78 | 0.72 | 0.226 | 396.30 |
| ψ(10k) | 402.83 | 0.77 | 0.221 | 396.35 |
| *M. pennanti* NATFRAG | | | | |
| ψ(10k) | 398.75 | 0.00 | 0.301 | 392.27 |
| ψ(5k) | 398.98 | 0.23 | 0.268 | 392.50 |
| ψ(1k) | 399.25 | 0.50 | 0.235 | 392.77 |
| ψ(0.5k) | 399.61 | 0.86 | 0.196 | 393.13 |
| *M. pennanti* PAVED | | | | |
| ψ(10k) | 400.40 | 0.00 | 0.415 | 393.92 |
| ψ(5k) | 400.81 | 0.41 | 0.338 | 394.33 |
| ψ(1k) | 402.82 | 2.42 | 0.124 | 396.34 |
| ψ(0.5k) | 402.83 | 2.43 | 0.123 | 396.35 |
| *M. americana* CON | | | | |
| ψ(10k) | 121.98 | 0.00 | 0.813 | 113.16 |
| ψ(5k) | 125.07 | 3.09 | 0.173 | 116.25 |
| ψ(1k) | 131.50 | 9.52 | 0.007 | 122.68 |
| ψ(0.5k) | 131.57 | 9.59 | 0.007 | 122.75 |
| *M. americana* ELE | | | | |
| ψ(10k) | 115.91 | 0.00 | 0.712 | 107.09 |
| ψ(5k) | 118.15 | 2.24 | 0.232 | 109.33 |
| ψ(1k) | 122.12 | 6.21 | 0.032 | 113.30 |
| ψ(0.5k) | 122.67 | 6.76 | 0.024 | 113.85 |
| *M. americana* FORCOV | | | | |
| ψ(10k) | 122.04 | 0.00 | 0.614 | 113.22 |
| ψ(5k) | 123.51 | 1.47 | 0.294 | 114.69 |
| ψ(1k) | 126.56 | 4.52 | 0.064 | 117.74 |
| ψ(0.5k) | 128.25 | 6.21 | 0.028 | 119.43 |
| *M. americana* NATFRAG | | | | |
| ψ(5k) | 125.21 | 0.00 | 0.375 | 116.39 |
| ψ(1k) | 126.09 | 0.88 | 0.241 | 117.27 |
| ψ(0.5k) | 126.53 | 1.32 | 0.194 | 117.71 |
| ψ(10k) | 126.57 | 1.36 | 0.190 | 117.75 |
| *M. americana* SNOW | | | | |
| ψ(5k) | 127.96 | 0.00 | 0.311 | 119.14 |
| ψ(10k) | 128.06 | 0.10 | 0.296 | 119.24 |
| ψ(1k) | 128.67 | 0.71 | 0.218 | 119.85 |
| ψ(0.5k) | 129.12 | 1.16 | 0.174 | 120.30 |
| *M. americana* TRI | | | | |
| ψ(10k) | 114.68 | 0.00 | 0.698 | 105.86 |
| ψ(5k) | 117.13 | 2.45 | 0.205 | 108.31 |
| ψ(0.5k) | 119.87 | 5.19 | 0.052 | 111.05 |
| ψ(1k) | 120.18 | 5.50 | 0.045 | 111.36 |
| *P. lotor* ELE | | | | |
| ψ(5k) | 318.02 | 0.00 | 0.273 | 309.20 |
| ψ(10k) | 318.22 | 0.20 | 0.247 | 309.40 |
| ψ(1k) | 318.23 | 0.21 | 0.245 | 309.41 |
| ψ(0.5k) | 318.31 | 0.29 | 0.236 | 309.49 |
| *P. lotor* FORCOV | | | | |
| ψ(10k) | 318.89 | 0.00 | 0.259 | 310.07 |
| ψ(5k) | 318.98 | 0.09 | 0.247 | 310.16 |
| ψ(0.5k) | 318.98 | 0.09 | 0.247 | 310.16 |
| ψ(1k) | 318.98 | 0.09 | 0.247 | 310.16 |
| *P. lotor* HOUSE | | | | |
| ψ(5k) | 316.69 | 0 | 0.3293 | 307.87 |
| ψ(1k) | 316.91 | 0.22 | 0.295 | 308.09 |
| ψ(0.5k) | 317.65 | 0.96 | 0.2038 | 308.83 |
| ψ(1k) | 317.99 | 1.3 | 0.1719 | 309.17 |
| *P. lotor* NATFRAG | | | | |
| ψ(1k) | 316.47 | 0 | 0.5209 | 307.65 |
| ψ(5k) | 318.69 | 2.22 | 0.1717 | 309.87 |
| ψ(10k) | 318.79 | 2.32 | 0.1633 | 309.97 |
| ψ(0.5k) | 319.04 | 2.57 | 0.1441 | 310.22 |
| *P. lotor* SHORE | | | | |
| ψ(1k) | 316.64 | 0 | 0.3722 | 307.82 |
| ψ(5k) | 317.11 | 0.47 | 0.2943 | 308.29 |
| ψ(10k) | 317.89 | 1.25 | 0.1992 | 309.07 |
| ψ(0.5k) | 318.68 | 2.04 | 0.1342 | 309.86 |
| *P. lotor* SNOW | | | | |
| ψ(10k) | 317.55 | 0 | 0.2937 | 308.73 |
| ψ(5k) | 317.9 | 0.35 | 0.2465 | 309.08 |
| ψ(1k) | 317.99 | 0.44 | 0.2357 | 309.17 |
| ψ(0.5k) | 318.09 | 0.54 | 0.2242 | 309.27 |
| *P. lotor* TRI | | | | |
| ψ(0.5k) | 315.47 | 0 | 0.3418 | 306.65 |
| ψ(1k) | 315.79 | 0.32 | 0.2913 | 306.97 |
| ψ(10k) | 316.71 | 1.24 | 0.1839 | 307.89 |
| ψ(5k) | 316.72 | 1.25 | 0.183 | 307.9 |
| *P. lotor* WETLAND | | | | |
| ψ(10k) | 317.92 | 0 | 0.3311 | 309.1 |
| ψ(5k) | 318.59 | 0.67 | 0.2369 | 309.77 |
| ψ(0.5k) | 318.73 | 0.81 | 0.2209 | 309.91 |
| ψ(1k) | 318.82 | 0.9 | 0.2111 | 310 |
| *Mustela* DEC | | | | |
| ψ(1k) | 92.35 | 0.00 | 0.889 | 83.53 |
| ψ(0.5k) | 97.60 | 5.25 | 0.064 | 88.78 |
| ψ(10k) | 99.62 | 7.27 | 0.024 | 90.80 |
| ψ(5k) | 99.67 | 7.32 | 0.023 | 90.85 |
| *Mustela* ELE | | | | |
| ψ(0.5k) | 104.62 | 0.00 | 0.252 | 95.80 |
| ψ(1k) | 104.62 | 0.00 | 0.252 | 95.80 |
| ψ(5k) | 104.65 | 0.03 | 0.248 | 95.83 |
| ψ(10k) | 104.65 | 0.03 | 0.248 | 95.83 |
| *Mustela* FORCOV | | | | |
| ψ(1k) | 92.69 | 0.00 | 0.787 | 83.87 |
| ψ(0.5k) | 96.21 | 3.52 | 0.135 | 87.39 |
| ψ(5k) | 98.21 | 5.52 | 0.050 | 89.39 |
| ψ(10k) | 99.38 | 6.69 | 0.028 | 90.56 |
| *Mustela* NATFRAG | | | | |
| ψ(10k) | 100.74 | 0.00 | 0.454 | 91.92 |
| ψ(5k) | 101.95 | 1.21 | 0.248 | 93.13 |
| ψ(1k) | 102.89 | 2.15 | 0.155 | 94.07 |
| ψ(0.5k) | 103.05 | 2.31 | 0.143 | 94.23 |
| *Mustela* SNOW | | | | |
| ψ(10k) | 98.21 | 0.00 | 0.770 | 89.39 |
| ψ(5k) | 101.55 | 3.34 | 0.145 | 92.73 |
| ψ(1k) | 103.90 | 5.69 | 0.045 | 95.08 |
| ψ(0.5k) | 104.12 | 5.91 | 0.040 | 95.30 |
| *Mustela* TRI | | | | |
| ψ(1k) | 101.05 | 0.00 | 0.336 | 92.23 |
| ψ(10k) | 101.21 | 0.16 | 0.310 | 92.39 |
| ψ(5k) | 101.39 | 0.34 | 0.283 | 92.57 |
| ψ(0.5k) | 104.15 | 3.10 | 0.071 | 95.33 |

^a^ Akaike Information Criterion for small samples

^b^ Model probability

^c^ Difference in -2Log(Likelihood) of the current model and -2log(Likelihood) of the saturated

model as a measure of model fit
