# Supplementary material for: Differential Habitat Use or Intraguild Interactions: What Structures a Carnivore Community?
Source: PLoS One. 2016 Jan 5;11(1):e0146055. doi: 10.1371/journal.pone.0146055 (PMC4711579; doi:10.1371/journal.pone.0146055)
Supplement: S5 Table — Cross-scale model selection results in the 90% confidence set estimating overall carnivore habitat occupancy in the Adirondack Mountains, New York. We fit encounter history data surveys at 54 sites in 2000–2002 to the candidate model set. For all models, probability of detection (p) was held as the most parsimonious model from stage 1 of modeling process for each species (S2 Table). Estimated occupancy (ψ) varied based combinations of local and larger-scale landscape and vegetative characteristics. Variable acronyms are in S1 Table. (DOCX) [file pone.0146055.s006.docx]

**Supporting Information**

**S5 Table. Cross-scale habitat models.** Cross-scale model selection results in the 90% confidence set estimating overall carnivore habitat occupancy in the Adirondack Mountains, New York. We fit encounter history data surveys at 54 sites in 2000–2002 to the candidate model set. For all models, probability of detection (*p*) was held as the most parsimonious model from stage 1 of modeling process for each species (S2 Table). Estimated occupancy (ψ) varied based combinations of local and larger-scale landscape and vegetative characteristics. Variable acronyms are in S1 Table.

| **Model** | **AIC_c_**^a^ | **ΔAIC_c_** | ***w***^b^ | **K**^c^ | **Deviance**^d^ |
| --- | --- | --- | --- | --- | --- |
| *U. americanus* | | | | | |
| ψ(BASNAG + FORCOV0.5k + LOGRD10k) | 250.36 | 0.00 | 0.185 | 7 | 233.93 |
| ψ(BASNAG + PAVED10k + LOGRD10k) | 251.04 | 0.68 | 0.132 | 7 | 234.61 |
| ψ(FORCOV0.5k + BASNAG + PAVED10k + LOGRD10k) | 251.05 | 0.69 | 0.131 | 8 | 231.85 |
| ψ(BASNAG + FORCOV0.5k+ DEC5k) | 251.29 | 0.93 | 0.116 | 7 | 234.86 |
| ψ(BASNAG + FORCOV0.5k + DEC5k + HEIGHT + CANOPEN) | 251.63 | 1.27 | 0.098 | 9 | 229.54 |
| ψ(BASNAG + PAVED10k + DEC5k) | 252.90 | 2.54 | 0.052 | 7 | 236.47 |
| ψ(LOGRD10k + NATFRAG10k) | 253.38 | 3.02 | 0.041 | 6 | 239.59 |
| ψ(FORCOV0.5k + BASNAG + PAVED10k) | 253.66 | 3.30 | 0.036 | 7 | 237.23 |
| ψ(BASNAG) | 254.00 | 3.64 | 0.030 | 5 | 242.75 |
| ψ(BASNAG + DEC5k) | 254.06 | 3.70 | 0.029 | 6 | 240.27 |
| ψ(BASNAG + PAVED10k + NATFRAG10k) | 254.63 | 4.27 | 0.022 | 7 | 238.20 |
| ψ(BASNAG + DEC5k + NATFRAG10k) | 255.00 | 4.64 | 0.018 | 7 | 238.57 |
| ψ(BASNAG + FORCOV0.5k) | 255.24 | 4.88 | 0.016 | 6 | 241.45 |
| *M. pennanti* | | | | | |
| ψ(NATFRAG10k + HOUSE5k) | 395.89 | 0.00 | 0.199 | 4 | 387.07 |
| ψ(HOUSE5k) | 395.90 | 0.01 | 0.198 | 3 | 389.42 |
| ψ(BASNAG + HOUSE5k) | 397.07 | 1.18 | 0.110 | 4 | 388.25 |
| ψ(PAVED10k + HOUSE5k) | 398.03 | 2.14 | 0.068 | 4 | 389.21 |
| ψ(NATFRAG10k + HOUSE5k + PAVED10k) | 398.15 | 2.26 | 0.064 | 5 | 386.90 |
| ψ(BASNAG + VOLCWD + HOUSE5k) | 398.19 | 2.30 | 0.063 | 5 | 386.94 |
| ψ(FORCOV0.5k + HOUSE5k) | 398.24 | 2.35 | 0.061 | 4 | 389.42 |
| ψ(NATFRAG10k) | 398.75 | 2.86 | 0.048 | 3 | 392.27 |
| ψ(NATFRAG10k + FORCOV0.5k) | 399.48 | 3.59 | 0.033 | 4 | 390.66 |
| ψ(FORCOV0.5k + HOUSE5k + BASNAG) | 399.49 | 3.60 | 0.033 | 5 | 388.24 |
| ψ(PAVED10k) | 400.40 | 4.51 | 0.021 | 3 | 393.92 |
| ψ(NATFRAG10k + CANOPEN) | 400.45 | 4.56 | 0.020 | 4 | 391.63 |
| *M. americana* | | | | | |
| ψ(VOLCWD + TRI10k) | 113.70 | 0.00 | 0.229 | 5 | 102.45 |
| ψ(TRI10k + BASNAG) | 114.45 | 0.75 | 0.157 | 5 | 103.20 |
| ψ(TRI10k) | 114.68 | 0.98 | 0.140 | 4 | 105.86 |
| ψ(TRI10k + NATFRAG5k) | 114.70 | 1.00 | 0.139 | 5 | 103.45 |
| ψ(TRI10k + CANOPEN) | 115.15 | 1.45 | 0.111 | 5 | 103.90 |
| ψ(FORCOV5k + TRI10k) | 116.98 | 3.28 | 0.044 | 5 | 105.73 |
| ψ(TRI10k + SNOW5k) | 117.04 | 3.34 | 0.043 | 5 | 105.79 |
| ψ(TRI10k + SNOW5k + ELE10k) | 117.59 | 3.89 | 0.033 | 6 | 103.80 |
| ψ(ELE10k + SNOW5k) | 118.26 | 4.56 | 0.023 | 5 | 107.01 |
| *P. lotor* | | | | | |
| ψ(TRI0.5k + HOUSE5k + dtHOUSE) | 312.90 | 0.00 | 0.290 | 6 | 299.11 |
| ψ(TRI0.5k + BASNAG + ASPECT0.5k) | 313.94 | 1.04 | 0.172 | 6 | 300.15 |
| ψ(TRI0.5k + BASNAG) | 314.04 | 1.14 | 0.164 | 5 | 302.79 |
| ψ(TRI0.5k + HOUSE5k + SHORE1k + BASNAG + dtHOUSE) | 314.12 | 1.22 | 0.158 | 8 | 294.92 |
| ψ(TRI0.5k + BASNAG + VOLCWD) | 316.38 | 3.48 | 0.051 | 6 | 302.59 |
| ψ(.) | 316.65 | 3.75 | 0.045 | 3 | 310.17 |
| ψ(TRI0.5k + HOUSE5k + SHORE1k + ASPECT0.5k + BASNAG + VOLCWD + dtHOUSE) | 317.74 | 4.84 | 0.026 | 10 | 292.62 |
| *Mustela* spp. | | | | | |
| ψ(DEC1k) | 92.35 | 0.00 | 0.279 | 4 | 83.53 |
| ψ(DEC1k + PROPSW) | 93.39 | 1.04 | 0.166 | 5 | 82.14 |
| ψ(DEC1k + SNOW10k) | 94.17 | 1.82 | 0.112 | 5 | 82.92 |
| ψ(VOLCWD + DEC1k + PROPSW) | 94.44 | 2.09 | 0.098 | 6 | 80.65 |
| ψ(DEC1k + VOLCWD) | 94.60 | 2.25 | 0.091 | 5 | 83.35 |
| ψ(DEC1k + CANOPEN) | 94.78 | 2.43 | 0.083 | 5 | 83.53 |
| ψ(FORCOV1k + ELE0.5k) | 95.09 | 2.74 | 0.071 | 5 | 83.84 |
| ψ(FORCOV1k + PROPSW) | 95.68 | 3.33 | 0.053 | 5 | 84.43 |

^a^ Akaike Information Criterion for small samples

^b^ Model probability

^c^ Number of model parameters

^d^ Difference in -2Log(Likelihood) of the current model and -2log(Likelihood) of the saturated

model as a measure of model fit
