# Supplementary material for: Differential Habitat Use or Intraguild Interactions: What Structures a Carnivore Community?
Source: PLoS One. 2016 Jan 5;11(1):e0146055. doi: 10.1371/journal.pone.0146055 (PMC4711579; doi:10.1371/journal.pone.0146055)
Supplement: S6 Table — Coefficient estimates (±SE) for variables by species based on cross-scale model selection results in the 90% confidence set estimating overall carnivore habitat occupancy in the Adirondack Mountains, New York. We fit encounter history data surveys at 54 sites in 2000–2002 to the candidate model set. Variable acronyms are in S1 Table. (DOCX) [file pone.0146055.s007.docx]

**Supporting Information**

**S6 Table. Coefficient estimates.** Coefficient estimates (±SE) for variables by species based on cross-scale model selection results in the 90% confidence set estimating overall carnivore habitat occupancy in the Adirondack Mountains, New York. We fit encounter history data surveys at 54 sites in 2000–2002 to the candidate model set. Variable acronyms are in S1 Table.

| Variable | *U. americanus* | *M. pennanti* | *M. americana* | *P. lotor* | *Mustela* spp. |
| --- | --- | --- | --- | --- | --- |
| HEIGHT | -0.16 (0.09) |  |  |  |  |
| VOLCWD |  | -0.04 (0.03) | 0.26 (0.16) | 0.02 (0.04) | 0.17 (0.28) |
| CANOPEN | -0.08 (0.08) | 0.01 (0.01) | -0.08 (0.06) |  | 0.28 (0.01) |
| BASNAG | 1.13 (0.59) | -0.09 (0.11) | 0.15 (0.10) | -0.46 (0.31) |  |
| PROPSW |  |  |  |  | 0.54 (0.33) |
| SHORE |  |  |  | -0.10 (0.08) |  |
| WATER |  |  |  |  |  |
| WETLAND |  |  |  |  |  |
| NATFRAG | 0.07 (0.06) | -0.26 (0.17) | 0.11 (0.07) |  |  |
| FORCOV | 0.66 (0.43) | 0.01 (0.01) | 0.01 (0.04) |  | 0.44 (0.27) |
| CON |  |  |  |  |  |
| DEC | -0.56 (0.32) |  |  |  | 3.10 (1.93) |
| dtLOGRD |  |  |  |  |  |
| dtPAVED |  |  |  |  |  |
| dtHOUSE |  |  |  | -0.58 (0.30) |  |
| HOUSE | -0.72 (0.18) | 2.59 (1.59) |  | -0.60 (0.29) |  |
| PAVED | -0.32 (0.22) | 0.02 (0.25) |  |  |  |
| LOGRD | 0.43 (0.24) |  |  |  |  |
| ASPECT |  |  |  | -0.12 (0.09) |  |
| ELE |  |  | 0.07 (0.06) |  | -0.10 (0.06) |
| TRI |  |  | 1.53 (0.57) | 0.98 (0.51) |  |
| SNOW |  |  | -0.01 (0.05) |  | 0.16 (0.22) |
